# Cardiovascular-kidney-metabolic health, genetic susceptibility, and incident cancer risk: a prospective UK biobank cohort and machine learning study

**DOI:** 10.3389/fendo.2026.1884752

**Published:** 2026-08-03

**Authors:** Xue He, Junqiao An, Chunjia Yan, Yutong Zhou, Ziyan Wu, Mengyao Xu, Qingyong He

**Affiliations:** 1 Graduate School, Beijing University of Chinese Medicine, Beijing, China; 2 Guang’anmen Hospital, China Academy of Chinese Medical Sciences, Beijing, China

**Keywords:** cancer, cardiovascular-kidney-metabolic syndrome, machine learning, prospective observational study, UK biobank

## Abstract

**Background:**

It remains unclear whether cardiovascular-kidney-metabolic (CKM) syndrome and genetic susceptibility are associated with cancer incidence. This study aims to evaluate the associations between CKM health status, genetic susceptibility, and cancer incidence, and to develop a machine learning-based prediction model for cancer risk in patients with advanced CKM syndrome.

**Methods:**

This study included 399,034 UK Biobank participants (389,289 with genetic data). CKM health was categorized into stages 0–4. Genetic susceptibility was assessed via a polygenic risk score (PRS). Associations were evaluated using Cox proportional hazard models. For participants with advanced CKM, candidate predictors were screened via the Boruta algorithm, LASSO regression, and multivariable logistic regression. Eight machine learning models were constructed and validated, and their predictive discrimination, calibration performance, and clinical practical value were further assessed. Additionally, Shapley Additive Explanations (SHAP) were adopted to interpret the internal mechanism of the optimal model. The dose–response relationship was assessed using restricted cubic splines (RCS), and mediation analysis was further performed to explore the underlying mechanism of the observed associations.

**Results:**

During a median follow-up of 13.7 years, 48,247 incident cancer cases were identified. Compared to CKM stage 0, multivariable-adjusted hazard ratios (HRs) were 1.01 (95% CI: 0.91-1.12) for stage 1, 1.21 (1.10–1.33) for stage 2, and 2.17 (1.95–2.42) for advanced CKM (stage 3–4). Participants with high PRS and advanced CKM faced the highest cancer risk (HR 3.24, 95% CI 2.68–3.93). A total of 14 predictors were retained after screening. The GBM model achieved the best predictive performance (test AUC = 0.801). A web-based calculator was developed to realize individualized cancer risk prediction. RCS analyses indicated that diastolic blood pressure (*P*_nonlinearity_<0.05), neutrophil count(*P*_nonlinearity_<0.05), alkaline phosphatase (*P*_nonlinearity_=0.001), and TyG-BMI (*P*_nonlinearity_<0.05) were significantly and nonlinearly associated with cancer risk. Total bilirubin the strongest positive mediating effect, explaining 44.45% of the total association (*P* < 0.001).

**Conclusions:**

Advanced CKM syndrome independently increases incident cancer risk, and this elevated risk is significantly amplified by high genetic susceptibility. The validated GBM model provides an interpretable tool for individualized risk estimation, which serves as a valuable complementary tool for clinical risk stratification and cancer patients with, cancer.

## 1 Introduction

A pioneering concept termed Cardiovascular-Kidney-Metabolic (CKM) syndrome was launched by the American Heart Association (AHA) in 2023. CKM syndrome refers to a collective group of multi-organ disorders resulting from the pathophysiological interactions among cardiovascular disease (CVD), chronic kidney disease (CKD), and metabolic risk factors. It is a complex clinical syndrome with diverse and complicated pathophysiological links involving multiple body systems (1). The AHA stratifies CKM into 0–4 stages: Stage 0, absence of CKM risks; Stage 1, abnormal adiposity; Stage 2, metabolic disorders or moderate-to-high-risk CKD; Stage 3, subclinical CVD; Stage 4, established clinical cardiovascular disease. This CKM staging structure reflects the pathophysiology, risk spectrum, and opportunities for optimizing prevention and care of CKM syndrome. The prevalence of CKM syndrome in the population is highly elevated; between 2011 and 2020, nearly 90% of US adults satified the diagnostic criteria for CKM syndrome (Stage 1 and above), among whom 15% are categorized as advanced-stage cases, and it was a primary cause of death in 2021 (2). CKM status significantly affects population morbidity and mortality. The clinical impact of poor CKM health is significant and leads to multisystem consequences. Nearly all major organ systems are impacted by CKM syndrome, accompanied by associated clinical challenges, including cardiovascular diseases (such as coronary heart disease, peripheral artery disease, and atrial fibrillation) (3–5), kidney failure (6), metabolic dysfunction-associated steatotic liver disease (7), premature cognitive decline (8, 9), and obstructive sleep apnea (10), among others.

Previous observational research has suggested that metabolic syndrome — a component of CKM syndrome—is linked to an elevated risk of cancer development (11–13). CVD and cancer frequently coexist, sharing multiple risk factors and pathophysiological mechanisms that may predispose patients to both conditions (14, 15). Furthermore, individuals with CVD tend to present with more risk factors, such as tobacco use, alcohol consumption, and obesity (16). These factors are associated with an increased release of inflammatory mediators and reactive oxygen species (ROS), which may contribute to DNA damage and be linked to cancer development. Currently, however, there are no prospective cohort studies investigating the association between CKM syndrome and cancer. The specific correlation between different CKM stages, particularly advanced CKM syndrome, and cancer incidence is still undefined, and the joint associations of genetic susceptibility require further investigation.

Given this background, the present study conducts a prospective cohort study based on the UK Biobank. While conventional epidemiological approaches, such as multivariable Cox proportional hazards models, excel at identifying independent, population-level risk factors, they inherently assume linear relationships and additive effects. However, CKM syndrome represents a highly complex multi-organ disorder characterized by interconnected pathophysiological networks, including systemic low-grade inflammation, metabolic shifts, and tissue cross-talk, which often are often characterized by non-linear trajectories and complex high-order interactions. To bridge this gap and maximize scientific value, this study introduces an framework that integrates genetic susceptibility (via polygenic risk scores, PRS) with multi-dimensional clinical, metabolic, and inflammatory biomarkers. Beyond establishing standard associative hazard ratios, we employ a comprehensive machine learning approach to capture complex non-linear interactions across these distinct genetic and phenotypic layers. This approach aims to transition from generalized population-level hazard identification to individualized, quantitative risk stratification, ultimately providing clinicians with an interpretable and actionable tool for early cancer screening and personalized preventative decision-making.

## 2 Methods

### 2.1 Study design and participants

Data for this study were acquired from the UK Biobank. Detailed information on the study design and methodology is available in prior studies (17, 18). Briefly, the UK Biobank is an extensive observational study launched in the United Kingdom in 2006, which enrolled over 500,000 participants aged 37 to 73 years at baseline. Sociodemographic characteristics, anthropometric measurements, medical history, biological samples, and imaging data were comprehensively and systematically collected using online questionnaires, face-to-face interviews, and clinical records. This study is a secondary analysis of de-identified data from the UK Biobank, a prospective cohort study with appropriate ethical approvals. The UK Biobank project received ethical approval from the National Health Service (NHS) National Research Ethics Committee (Approval No.: 21/NW/0157), and all participants provided written informed consent prior to study participation. The study was carried out under UK Biobank application number 906426. We formally declare that all statistical and machine learning analyses were strictly conducted within the UK Biobank Secure Analysis Platform, and no individual-level data were exported at any stage. Furthermore, the data have not been transferred to any third party, nor have they been utilized for any commercial purposes.

The initial cohort for this study comprised 501,936 participants, from which 25,303 individuals diagnosed with cancer at baseline were excluded. Additionally, participants with incomplete data on CKM-related variables (N = 75,589), with missing information on potential covariates (N = 484) or who were lost to follow-up (N = 1,526) were also excluded. To evaluate the potential for selection bias introduced by these exclusions, a comparative analysis of baseline characteristics was conducted between the enrolled cohort and the participants with incomplete clinical data. Ultimately, A total of 399,034 participants were enrolled in the primary analysis. To control for population heterogeneity, the genetic analysis was restricted to 389,289 individuals with complete genetic data. **Supplementary Figure 1** presents the flowchart of participant screening and enrollment.

### 2.2 Classification of CKM syndrome stages

Baseline CKM syndrome staging (Stages 0 to 4) was defined based on the AHA criteria, with specific adaptations for the UK Biobank (1). Stage 0 was defined as having a body mass index (BMI) between 18.5 and 24.9, a waist circumference of less than 88 cm in females or less than 102 cm in males, and no other risk factors. Stage 1 was assigned to participants presenting at least one of the following conditions: (i) BMI ≥ 25 kg/m²; (ii) elevated waist circumference (≥ 88 cm in females, ≥ 102 cm in males); or (iii) glycated hemoglobin A1c (HbA1c) concentrations ranging from 39 to 46 mmol/mol. Stage 2 covered subjects with established metabolic abnormalities, including hypertriglyceridemia (≥ 1.7 mmol/L), hypertension, diabetes mellitus, metabolic syndrome (MetS), as well as those with moderate-to-high CKD risk defined by KDIGO criteria. Stage 3 consisted of individuals with very high CKD risk or an elevated 10-year predicted CVD risk. Stage 4 included participants with a prior CVD diagnosis, such as coronary artery disease, atrial fibrillation, heart failure, stroke, and peripheral arterial disease. Advanced CKM syndrome was collectively defined as Stage 3 and Stage 4. Consistent with the American Heart Association (AHA) guidelines, CKM syndrome Stage 3 and Stage 4 were considered as advanced CKM syndrome in our primary analyses. Pathophysiologically, Advanced CKM possesses a distinct clinical integrity: while Stages 1–2 represent the accumulation of subclinical metabolic risk factors, Stages 3–4 mark a critical qualitative shift into established target organ damage or overt clinical cardiovascular disease. From a statistical perspective, this consolidation also served to mitigate potential data sparsity in CKM stage 3-4. Detailed diagnostic criteria for all relevant risk factors and clinical outcomes are summarized in **Supplementary Table 1**.

### 2.3 Assessment of cancer

The National Health Service Central Register provided cancer diagnostic information to the UK Biobank. Detailed information regarding cancer was obtained through the United Kingdom and Ireland Association of Cancer Registries. Cancer cases were confirmed using the ICD-10 code “C00-C97”. Participants were followed up from baseline to the earliest occurrence of cancer diagnosis, death, or the administrative end of follow-up. To minimize potential reverse causation bias in the primary analysis, participants who developed incident cancer within 1 year after baseline were excluded from the primary analytical cohort. Detailed descriptions of the ICD-10 codes for cancer and other diseases evaluated in this study are provided in **Supplementary Table 2**.

### 2.4 Covariates

Data regarding sociodemographic characteristics and lifestyle behaviors was self-reported via touchscreen questionnaires, including sex (male, female), age (years), ethnicity (White, non-White), current alcohol consumption status (yes, no), current smoking status (yes, no), and family history of cancer (yes or no). Socioeconomic status (Townsend deprivation index) was assessed based on participants’ postal codes, with higher scores indicating greater levels of deprivation. Physical activity was expressed as the sum of metabolic equivalent (MET) time per week for all activities. Detailed information of the covariates are provided in **Supplementary Table 3**.

### 2.5 Genetic risk assessment

We selected single nucleotide polymorphisms (SNPs) at the genome-wide significance level as predictors to construct the polygenic risk score (PRS), comprising 102 cancer-associated SNPs. These genetic variants and their corresponding coefficient estimates were derived from large-scale European genome-wide association studies (GWAS) targeting generalized malignant neoplasms (**Supplementary Table 4**). Only variants demonstrating genome-wide significance for cancer with a minor allele frequency exceeding 1% were retained. To ensure the mutual independence of these genetic variants, linkage disequilibrium (LD) clumping was performed based on a European reference panel to eliminate variants in high LD. For each participant, the cancer PRS was computed by multiplying the dosage of risk alleles (0, 1, or 2) at each locus by its respective regression coefficient, followed by an aggregation of these weighted values. The formula of calculating the PRS is as follows:


$$PRS=\sum_{i-1}^{n}\;(\beta i\times genotype_{i})$$

Where *n* represents the number of cancer-associated SNPs considered. *βi* is the effect size of the *i*th SNP, typically expressed as the natural logarithm of the odds ratio (OR). $genotype_{i}$ represents the genotype of the *i*th SNP, determined by whether the individual carries the risk allele. These data were all derived from published GWAS. We calculated the PRS by multiplying the number of each risk allele by its corresponding disease effect size (log[OR]) and summing the products. Based on the ordered tertiles of the PRS distribution, participants were subsequently stratified into three genetic risk categories: Low, Moderate, and High.

### 2.6 Machine learning to predict association

In the initial association analysis, we observed that individuals with advanced CKM (Stages 3-4) faced a substantially elevated risk of incident cancer relative to the Stage 0 baseline. Consequently, we constructed a machine learning-based prediction model for cancer risk specifically targeting the population with CKM Stages 3-4. To ensure a rigorous validation protocol and completely prevent data leakage, the complete dataset was first randomly partitioned into a 70% training subset and a 30% validation subset before any data preprocessing, resampling, or feature engineering was conducted. Given the extraordinarily large sample size of our cohort, reliance solely on traditional significance tests may erroneously inflate negligible variances into statistically significant p-values. Therefore, standardized mean differences (SMDs) were calculated alongside p-values to evaluate baseline comparability between the training and validation datasets, with an SMD < 0.10 indicating negligible imbalance. Within the training subset, the Synthetic Minority Oversampling Technique (SMOTE) was exclusively applied to mitigate class imbalance and enhance the model’s risk stratification capability for the minority outcome category, while the validation subset remained entirely untouched to preserve its real-world clinical distribution. For feature engineering within the balanced training cohort, Spearman’s rank correlation coefficient and the variance inflation factor (VIF) were first employed to assess multicollinearity among the 62 candidate variables (**Supplementary Table 5**); variables exhibiting a VIF greater than 5 were strictly excluded. Subsequently, a sequential feature selection workflow was implemented on the remaining pool: First, the Boruta algorithm was utilized to isolate variables of high importance by comparing original features against permuted shadow features via a random forest model. Second, Least Absolute Shrinkage and Selection Operator (LASSO) regression was applied to further penalize non-essential features, shrinking their coefficients to zero via an L1 regularization penalty. Third, multivariable logistic regression using a stepwise selection approach based on the likelihood ratio test was conducted to lock the final core predictors. The validation subset was utilized solely for the final, unbiased performance evaluation of the optimized model.

Subsequently, we constructed and evaluated eight machine learning models: Adaptive Boosting (AdaBoost), Categorical Boosting (CatBoost), eXtreme Gradient Boosting (XGBoost), Gradient Boosting Machine (GBM), Logistic Regression, Light Gradient Boosting Machine (LightGBM), Neural Network, and Support Vector Machine (SVM). To optimize model performance while preventing overfitting, hyperparameters for each algorithm were rigorously tuned using a grid-search strategy coupled with 5-fold internal cross-validation exclusively within the training dataset. To guarantee the clinical utility and generalization stability of the deployed predictive tool, standard performance benchmarks for final model selection were pre-specified. Specifically, candidate algorithms exhibiting substantial generalization decay (defined as an excessive absolute discrepancy in AUC between the training and validation sets) or an unacceptable drop in clinical sensitivity on the unseen testing data were systematically precluded from final adoption. Final model performance was evaluated on the independent validation subset using Receiver Operating Characteristic (ROC) curves and the Area Under the Receiver Operating Characteristic Curve (AUC). It was further comprehensively interpreted via performance metrics including threshold, accuracy, sensitivity, specificity, and precision. The DeLong test was employed to determine whether there were statistically significant differences in predictive performance among the different models. Decision curve analysis (DCA) was applied to quantify the net clinical benefit at different threshold probabilities, and calibration curves were adopted to evaluate the consistency between model-predicted risks and actual observed outcomes.

Shapley Additive Explanations (SHAP) analysis via the R package shapviz was performed to interpret the predictive outputs of the final optimal model. Concurrently, the importance ranking of the features in the final model was calculated. Finally, the prediction model was deployed via a web interface to provide an accessible, visual application.

### 2.7 Statistical analyses

Participants’ baseline characteristics were expressed as mean ± SD for continuous variables and n (%) for categorical variables. Cumulative outcome incidence was determined using the Kaplan–Meier method (survminer, survival, and ggplot2 in R). Cox proportional hazards models were utilized to estimate hazard ratios (HRs) and 95% confidence intervals (CIs), with CKM Stage 0 as the reference. Schoenfeld residuals confirmed no violations of the proportional hazards assumption. Three adjusted models were analyzed: Model 2 controlled for age, sex, and ethnicity; Model 3 added adjustments for smoking, alcohol consumption, physical activity, Townsend deprivation index, and family history of cancer. All participant data were systematically collected through standardized questionnaires, interviews, and clinical documentation, and written informed consent was obtained prior to study participation.

To explore the joint association of CKM status and genetic risk, an interaction term (CKM syndrome stage × cancer PRS) was incorporated into the multiple-adjusted model. Furthermore, to analyze the potential joint associations of genetic risk and CKM syndrome, a 12-level categorical variable was constructed, encompassing the 12 combinations of the cancer PRS categories (Low, Moderate, and High) and CKM syndrome stages (Stage 0, Stage 1, Stage 2, and Stages 3-4). The subgroup comprising individuals with a “Low cancer PRS and CKM syndrome Stage 0” served as the reference group for this analysis.

We utilized Cox proportional hazards models to perform subgroup analyses, stratified by age, sex, ethnicity, and other relevant covariates. To explore the interaction between CKM syndrome and these covariates, interaction terms between CKM status and the covariates were incorporated into the main model. In sensitivity analysis, the lag-time window was extended to 2 years (versus 1 year in the primary analysis) to strictly account for potential subclinical or undiagnosed malignancies. R software (version 4.4.3) was used for all statistical analyses, and statistical significance was defined as a *P*-value < 0.05.

For the eight continuous variables selected via LASSO, Boruta, and multivariable logistic regression, namely diastolic blood pressure (DBP), total bilirubin (TBIL), alanine aminotransferase (ALT), alkaline phosphatase (ALP), neutrophil (NE), C-reactive protein-triglyceride glucose index (CTI), triglyceride-glucose body mass index (TyG-BMI), and potassium intake—restricted cubic spline (RCS) and multivariable mediation analyses were advanced to decode their detailed epidemiological roles and clinical implications. First, to transition from empirical prediction to actionable clinical boundaries, RCS models (with 3 knots) were employed to test for non-linear trends, thereby analyzing dose-response relationships and facilitating mediation analyses and identifying critical clinical risk thresholds. Curves were plotted to visualize threshold effects, and likelihood ratio tests were used to compare the goodness-of-fit between linear and non-linear models; a P-value < 0.05 indicated a significant non-linear association. Second, to quantify the extent to which these top-tier predictors account for the observed risk transition, these eight variables were evaluated as potential mediators within a multivariable mediation framework. Analyses were estimated using the bootstrapping method with 1,000 resamples to evaluate their specific indirect statistical effects (whether reinforcing or counteracting) on the overall association between baseline CKM syndrome status and incident cancer risk. This generated indirect effect estimates, standard errors (SE), and corresponding P-values.

## 3 Result

### 3.1 Explore association

#### 3.1.1 Baseline characteristics of the study population

**Supplementary Figure 1** illustrate the study flow. A total of 399,034 participants were included in this study, with a mean age of 56.63 ± 8.08 years, and 53.73% were female. To evaluate potential selection bias, a comprehensive comparison of baseline characteristics between these included participants and those excluded due to incomplete CKM-related data ( n=75,589) was performed, demonstrating a highly balanced distribution between the two populations (**Supplementary Table 6**). At baseline, 30,394 (7.62%) participants were classified as stage 0, 134,786 (33.78%) as stage 1, 191,192 (47.91%) as stage 2, and 42,662 (10.69%) as stages 3–4 of CKM syndrome. **Table 1** presents the distribution of participant characteristics across the different CKM status. As the CKM syndrome stage progressed, participants tended to be older, the proportion of males increased, and the prevalence of health risk factors, such as smoking and a family history of cancer, was higher. Participants with advanced CKM syndrome (Stages 3–4) tended to have lower household income and educational attainment, along with greater waist circumference and higher BMI. Furthermore, these participants exhibited declined renal function (manifested by a lower estimated glomerular filtration rate [eGFR]) and metabolic abnormalities (manifested by higher glycated hemoglobin [HbA1c], fasting blood glucose, systolic blood pressure, and diastolic blood pressure).

**Table 1:** Baseline characteristics of the study population.

Characteristics	Total	CKM			
		Stage 0	Stage 1	Stage 2	Stage 3–4
Number of participants, n (%)	399,034	30,394 (7.62)	134,786 (33.78)	191,192 (47.91)	42,662 (10.69)
Sociodemographic factors					
Age at recruitment (years)	56.63 (8.08)	53.85 (8.11)	55.69 (8.07)	56.83 (7.78)	63.04 (5.72)
Female, n (%)	208,843 (52.34)	21,565 (70.95)	81,290 (60.31)	96,418 (50.43)	9,570 (22.43)
White ethnicity, n (%)	353,449 (88.58)	27,078 (89.09)	118,065 (87.59)	169,989 (88.91)	38,317 (89.82)
Townsend deprivation index	-1.29 (3.09)	-1.58 (2.94)	-1.35 (3.07)	-1.26 (3.10)	-0.82 (3.31)
College or university degree, n (%)	125,427 (31.43)	13,133 (43.21)	45,324 (33.63)	57,836 (30.25)	9,134 (21.41)
Income before tax >52,000 EUR, n (%)	95,693 (23.98)	9,896 (32.56)	36,417 (27.02)	43,974 (23.00)	5,406 (12.67)
Lifestyle information					
BMI (kg/m^2^)	27.65 (4.79)	22.57 (1.68)	27.89 (3.91)	28.81 (4.89)	28.99 (4.82)
Waist circumference (cm)	90.90 (13.39)	76.54 (7.66)	89.82 (11.29)	94.51 (12.74)	98.44 (12.73)
Current smoker, n (%)	43,374 (10.87)	2,632 (8.66)	11,432 (8.48)	19,406 (10.15)	9,904 (23.22)
Current drinking, n (%)	366,168 (91.76)	28,494 (93.75)	125,217 (92.90)	174,463 (91.25)	37,994 (89.06)
Physical activity (MET min/week)	2644.91 (2656.69)	2798.06 (2640.30)	2708.16 (2668.07)	2559.32 (2635.60)	2656.27 (2742.83)
Family history of cancer, n (%)	177,208 (44.41)	12,334 (40.58)	58,862 (43.67)	85,750 (44.85)	20,262 (47.49)
Laboratory measurements					
Blood glucose (mmol/L)	5.14 (1.26)	4.77 (0.70)	5.05 (0.92)	5.22 (1.38)	5.55 (1.93)
HbAlc (mmol/mol)	36.22 (6.58)	33.45 (3.68)	35.37 (5.19)	36.92 (7.30)	39.74 (10.01)
Total Cholesterol (mmol/L)	5.70 (1.15)	5.55 (0.98)	5.62 (1.05)	5.88 (1.18)	5.16 (1.31)
Triglycerides (mmol/L)	1.77 (1.04)	0.98 (0.27)	1.18 (0.53)	2.29 (1.04)	2.10 (1.22)
HDL-C (mmol/L)	1.44 (0.38)	1.71 (0.38)	1.56 (0.38)	1.33 (0.33)	1.24 (0.32)
LDL-C (mmol/L)	2.60 (0.74)	2.51 (0.64)	2.61 (0.69)	2.68 (0.77)	2.26 (0.81)
Systolic blood pressure (mmHg)	138.23 (18.65)	130.06 (17.72)	136.35 (17.91)	139.89 (17.24)	148.70 (22.72)
Diastolic blood pressure (mmHg)	82.44 (10.14)	77.90 (9.52)	82.00 (9.72)	83.84 (9.84)	83.72 (11.66)

Data are shown as mean (SD) for continuous variables and number (%) for categorical variables. Percentages might not add up to 100% due to rounding.

#### 3.1.2 Association of CKM syndrome stages with incident cancer

During a median follow-up of 13.7 years (interquartile range [IQR]: 13.05–14.44 years), a total of 48,247 incident cancer cases were identified. As shown in **Supplementary Figure 2**, the cumulative incidence of cancer was significantly higher in stages 3–4 compared to the earlier stages. **Table 2** presents the associations between the different CKM syndrome stages and the risk of incident cancer. Compared with stage 0, the unadjusted HRs (95% CIs) for developing cancer in patients with CKM stage 1, stage 2, and advanced CKM were 1.19 (1.07, 1.32) (*P* < 0.05), 1.58 (1.43, 1.74) (*P* < 0.001), and 4.57 (4.13, 5.06) (*P* < 0.001), respectively. After adjusting for potential confounders, the HRs (95% CIs) for incident cancer in patients with CKM stage 1, stage 2, and advanced CKM were 1.01 (0.91, 1.12) (*P* = 0.92), 1.21 (1.10, 1.33) (*P* < 0.001), and 2.17 (1.95, 2.42) (*P* < 0.001), respectively. A dose-response association between the CKM syndrome stage and cancer was observed in the multivariable-adjusted model [HR: 1.32 (1.27, 1.36), *P* < 0.001] (**Table 2**).

**Table 2:** The joint association of CKM syndrome and polygenic risk scores with risk of cancer.

Exposure	N	Cases	Model 1 HR (95% CI)	*P*	Model 2 HR (95% CI)	*P*	Model 3 HR (95% CI)	*P*
Association of CKM syndromestage and risk of cancer								
Stage 0	30,394	2,132	**Ref**		**Ref**		**Ref**	
Stage 1	134,786	11,251	1.19 (1.07, 1.32)	0.001	1.05 (0.94, 1.16)	0.396	1.01 (0.91, 1.12)	0.92
Stage 2	191,192	21,189	1.58 (1.43, 1.74)	<0.001	1.29 (1.17, 1.42)	<0.001	1.21 (1.1, 1.33)	<0.001
Stage 3-4	42,662	13,675	4.57 (4.13, 5.06)	<0.001	2.51 (2.26, 2.8)	<0.001	2.17 (1.95, 2.42)	<0.001
Joint association of CKM syndrome and PRS with risk of cancer								
Stage 0+Low PRS	10,317	559	**Ref**		**Ref**		**Ref**	
Stage 0+Moderate PRS	10,183	641	1.16 (0.92, 1.47)	0.2	1.19 (0.95, 1.5)	0.133	1.18 (0.94, 1.49)	0.153
Stage 0+High PRS	9,819	932	1.75 (1.41, 2.16)	<0.001	1.86 (1.5, 2.31)	<0.001	1.81 (1.46, 2.24)	<0.001
Stage 1+Low PRS	43,649	2,744	1.16 (0.95, 1.43)	0.152	1.01 (0.82, 1.24)	0.909	0.99 (0.8, 1.21)	0.898
Stage 1+Moderate PRS	43,571	3,332	1.41 (1.15, 1.72)	0.001	1.27 (1.04, 1.55)	0.019	1.21 (0.99, 1.47)	0.062
Stage 1+High PRS	43,427	4,450	1.89 (1.56, 2.29)	<0.001	1.77 (1.46, 2.14)	<0.001	1.65 (1.36, 2)	<0.001
Stage 2+Low PRS	63,581	6,103	1.77 (1.48, 2.13)	<0.001	1.45 (1.21, 1.74)	<0.001	1.38 (1.15, 1.65)	0.001
Stage 2+Moderate PRS	63,617	6,520	1.89 (1.57, 2.26)	<0.001	1.57 (1.31, 1.89)	<0.001	1.47 (1.22, 1.76)	<0.001
Stage 2+High PRS	63,703	8,410	2.44 (2.04, 2.91)	<0.001	2.12 (1.78, 2.54)	<0.001	1.92 (1.6, 2.29)	<0.001
Stage 3-4+Low PRS	12,216	3,305	4.99 (4.1, 6.07)	<0.001	2.74 (2.24, 3.34)	<0.001	2.43 (1.99, 2.97)	<0.001
Stage 3-4+Moderate PRS	12,392	3,884	5.78 (4.77, 7)	<0.001	3.22 (2.65, 3.91)	<0.001	2.78 (2.29, 3.38)	<0.001
Stage 3-4+High PRS	12,814	4,745	6.83 (5.66, 8.24)	<0.001	3.92 (3.24, 4.75)	<0.001	3.24 (2.68, 3.93)	<0.001

These models utilized the Cox proportional hazards model. Model 1 was unadjusted. Model 2 was adjusted for age, sex, ethnic. Model 3 was adjusted for age, sex, ethnic, Townsend, family history of cancer, physical activity, drinking and smoking.

Models were constructed using Cox proportional hazards regression. Model 1 was unadjusted. Model 2 was adjusted for age, sex, and ethnicity. Model 3 was fully adjusted for age, sex, ethnicity, smoking status, alcohol consumption, physical activity, Townsend deprivation index, and family history of cancer. To evaluate the combined impact of clinical phenotyping and genetic susceptibility, a 12-level joint categorical variable was constructed by cross-stratifying the four CKM categories (Stage 0, 1, 2, and 3–4) with the three PRS tertiles (Low, Moderate, and High). Participants characterized by both a low cancer PRS and CKM Stage 0 served as the reference group. HR, hazard ratio; CI, confidence interval; CKM, cardiovascular-kidney-metabolic; PRS, polygenic risk score; Ref, reference category.

#### 3.1.3 Joint associations of genetic susceptibility and CKM stages

Compared with individuals with a low cancer PRS, the fully adjusted HRs for incident cancer among individuals with intermediate and high PRS in the multivariable model were 1.13 (95% CI: 1.05–1.21, *P* < 0.05) and 1.48 (95% CI: 1.38–1.58, *P* < 0.001), respectively. In the model investigating the joint associations of genetic risk for cancer and CKM health, a significant interaction was observed between the CKM syndrome stage and cancer PRS (*P* for interaction < 0.001). Compared to individuals with a low cancer PRS and CKM syndrome stage 0, those with a high cancer PRS and CKM syndrome stages 3–4 exhibited the highest risk of cancer (HR = 3.24, 95% CI: 2.68–3.93) (**Table 2**).

#### 3.1.4 Subgroup and sensitivity analysis

**Figure 1:**
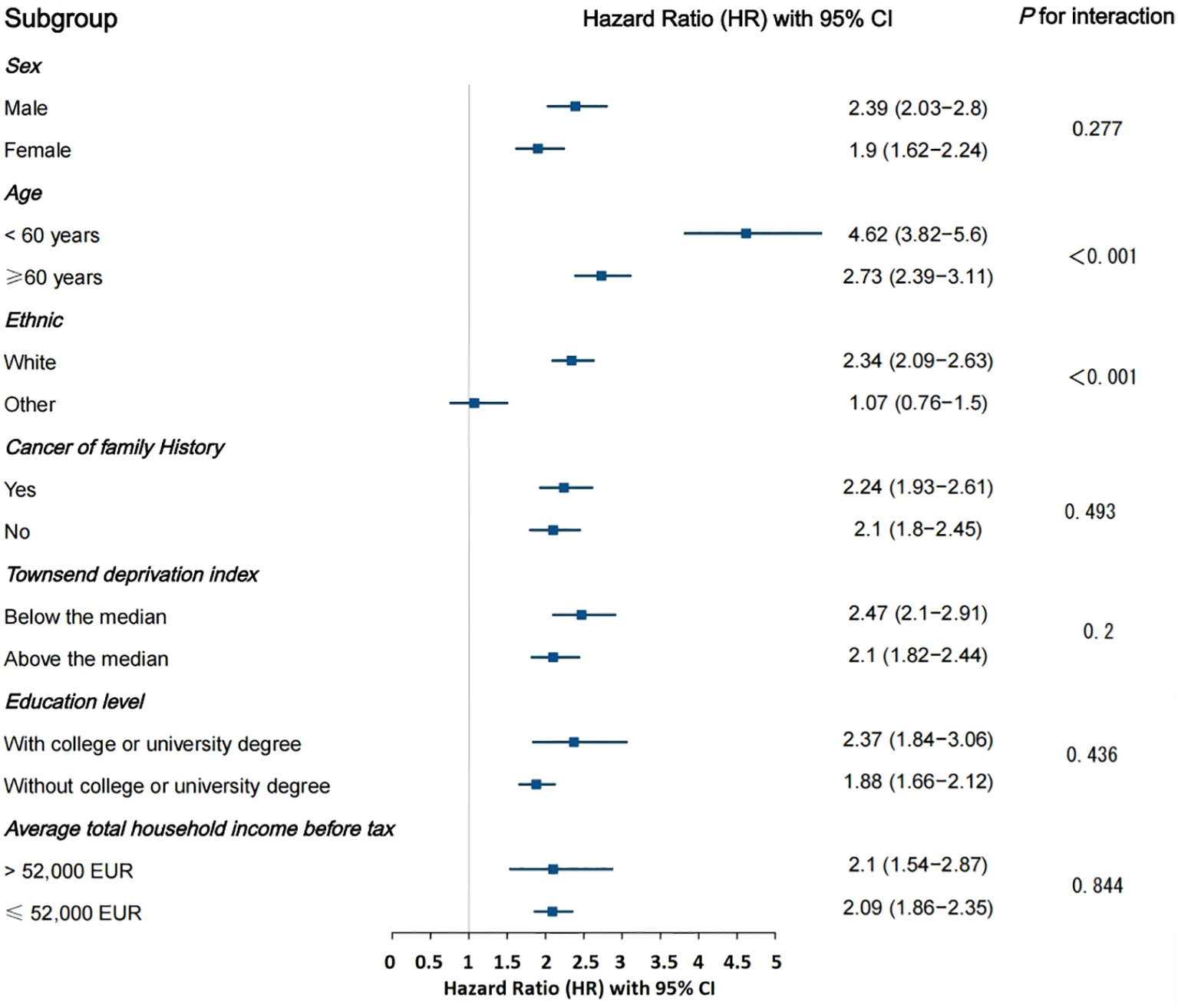
Subgroup analysis of the association between CKM health and the risk of incident cancer. Forest plot showing the HR and 95% CI across various subgroups. The blue squares indicate the point estimates of the HR, and the horizontal lines denote the corresponding 95% CI. The vertical solid gray line represents the reference value of no effect (HR = 1.0). The P for interaction assesses whether the magnitude of the association differs significantly across the strata of each subgroup. Significant interactions were observed for age (*P* < 0.001) and ethnic (*P* < 0.001).

In subgroup analyses based on sex, family history of cancer, Townsend deprivation index, educational level, and income level, these associations remained robust (**Figure 1**). Significant interactions were observed between CKM syndrome and both age and race. The association between CKM syndrome and cancer risk was more pronounced among participants aged < 60 years (< 60 years, HR: 4.62, 95% CI: 3.82–5.60). Similarly, the association between CKM syndrome and cancer risk was more prominent in White participants (White, HR: 2.34, 95% CI: 2.09–2.63) (**Figure 1**). To evaluate the robustness of the analyses, we conducted a sensitivity analysis by excluding incident cancer cases that occurred within the first 2 years of follow-up to minimize potential reverse causality. The trend of the association between CKM health and cancer remained consistent (**Supplementary Table 7**).

### 3.2 Machine learning-based risk prediction

#### 3.2.1 Feature selection and variable identification

**Figure 2:**
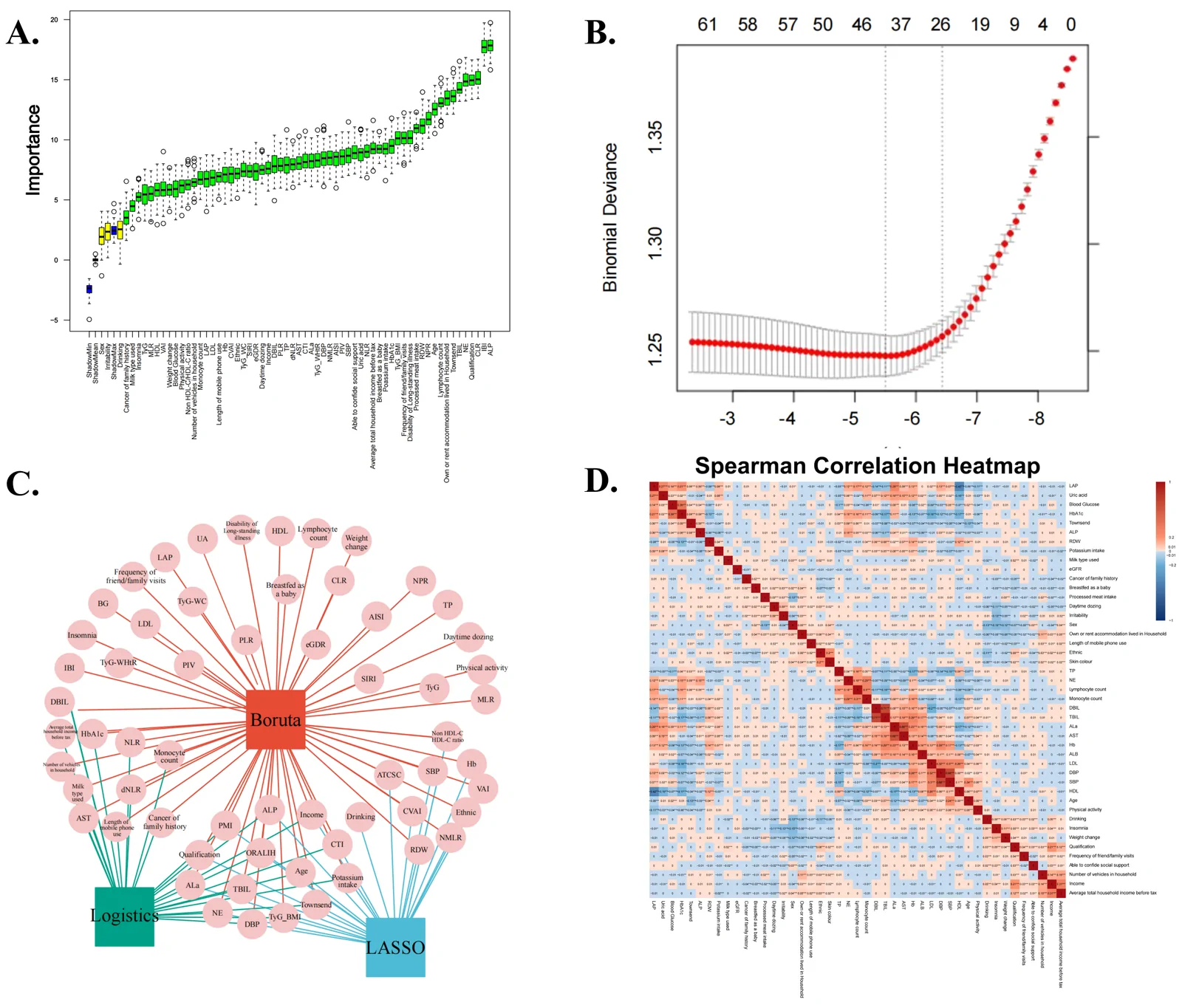
Machine-learning–based feature selection. **(A)** Feature importance identifed by the Boruta algorithm (green: confirmed, yellow: tentative). **(B)** Coefficient paths of LASSO regression across varying log(lambda) values. **(C)** common predictors between Boruta, Logistics and LASSO. **(D)** Spearman correlation heatmaps of candidate variables in the training cohort. Color denotes correlation strength and direction (blue: positive, red: negative); darker shades represent stronger correlations.

The baseline demographics and clinical profiles were highly comparable between the training dataset (70%) and the testing dataset (30%). Reflecting the very large sample size. All calculated SMDs were lower than 0.1 and p-value were higher than 0.05, confirming an optimal equilibrium for the baseline characteristics (**Supplementary Table 8**). To identify the key predictors, this study combined three feature selection methods. Among the 62 included variables, the Boruta algorithm did not demonstrate a clear differentiation (**Figure 2**). Subsequently, LASSO regression and multivariable logistic regression analysis were utilized to further screen and identify the critical independent variables (**Supplementary Table 9**). Spearman correlation analysis demonstrated low-to-moderate correlations among all included variables (all |r| < 0.5). The potential influence of multicollinearity was excluded using the variance inflation factor (all VIFs < 5) (**Figure 2**; **Supplementary Table 10**). By taking the intersection of the features derived from the three selection methods, 14 core predictors were ultimately retained: Processed meat intake, alkaline phosphatase, own or rent accommodation lived in, NE, age, TyG_BMI, TBIL, Townsend deprivation index, qualifications, CTI, potassium intake, DBP, ALa and income. These predictors were subsequently incorporated into the construction of the joint model (**Figure 2**).

#### 3.2.2 Model performance and validation assessment

**Figure 3:**
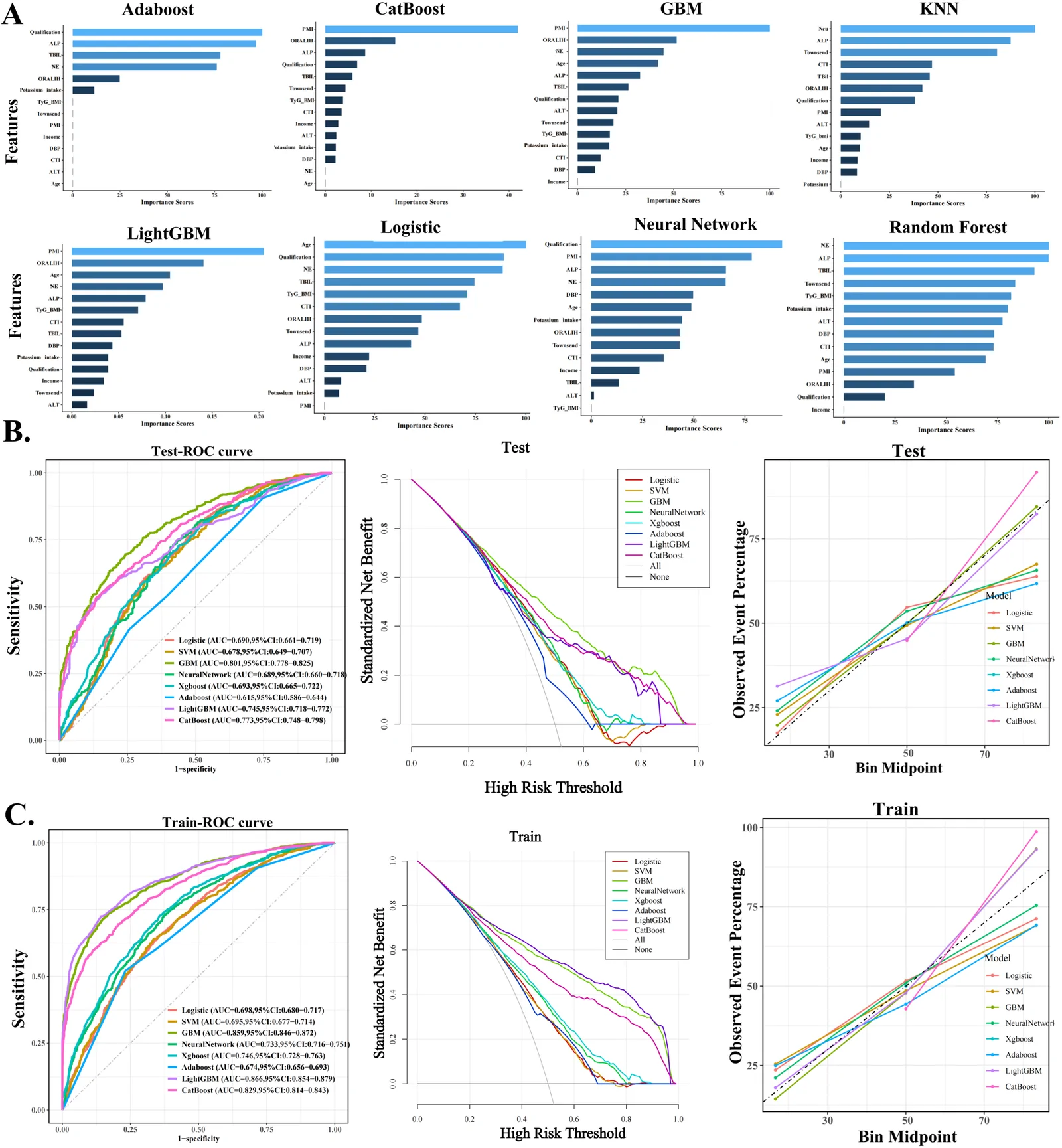
Feature importance rankings and model performance of machine learning models for predicting cancer incidence among advanced CKM syndrome. **(A)** Top-ranked predictors across different machine learning models. The figure depicts the ten most influential features identifed by eight machine learning algorithms: Adaboost, CatBoost, GBM, KNN, LightGBM, Logistic, neural network (NN) and support vector machine (SVM). Feature importance was derived using metrics specific to each model (e.g., standardized coefficients for LR, Gini importance for RF). **(B)** ROC curves, DCA and Calibration plots of Test dataset. **(C)** ROC curves, DCA and Calibration plots of Train dataset.

Eight supervised machine learning algorithms were applied to develop predictive models for incident cancer among individuals with CKM stage 0-3. The candidate algorithms included AdaBoost, CatBoost, XGBoost, GBM, Logistic Regression, LightGBM, Neural Network, and SVM. The GBM model demonstrated optimal performance, achieving an AUC of 0.801 (95% CI: 0.778–0.825) in the testing set, and achieved the highest accuracy (0.727), sensitivity (0.695), specificity (0.758), precision (0.742), and F1 score (0.718) (**Figure 3**; **Supplementary Table 11**). Decision curve and calibration curve analyses further confirmed that this model provided the maximum net clinical benefit across the entire range of probability thresholds, along with high predictive accuracy. The GBM model also exhibited robust performance in the training set, with an AUC of 0.859 (95% CI: 0.846–0.872) (**Figure 3**). The confusion matrix for the GBM model indicated a favorable balance between sensitivity and specificity (**Figure 3**). Furthermore, the DeLong test confirmed significant differences in discriminative performance between the GBM and the other models (**Supplementary Table 12**). Regarding candidate model selection, LightGBM was not adopted due to its failure to satisfy the stringent generalization and clinical safety thresholds. Although LightGBM yielded the highest discriminative capacity during the training phase (AUC = 0.866), its performance exhibited substantial optimization decay upon validation, with the testing AUC decreasing to 0.745. Crucially, its testing sensitivity plummeted to an inadequate 0.547, indicating a high risk of false negatives. Consequently, based on a data-driven evaluation prioritizing robust generalization stability and balanced performance on unseen data, LightGBM was filtered out during the algorithmic screening process, further validating the GBM model as the final optimal and most stable predictive framework.

#### 3.2.3 Model interpretability and interactive application

**Figure 4:**
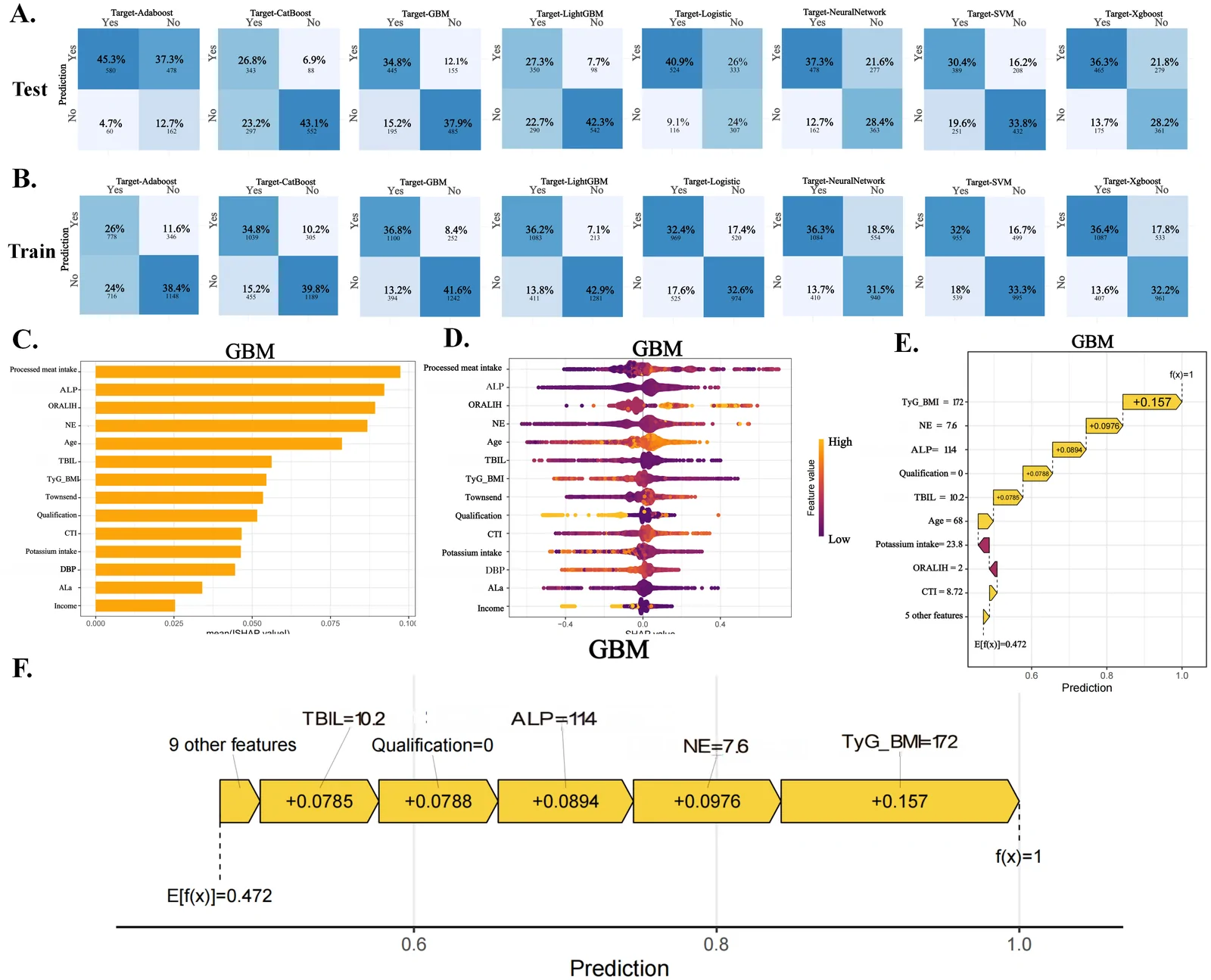
Confusion matrices and SHAP analysis for predicting cancer incidence among advanced CKM syndrome. **(A)** Confusion matrices of six machine learning models across Test dataset. **(B)** Confusion matrices of six machine learning models across Train dataset. **(C)** SHAP bar plot displaying mean absolute SHAP values, representing global feature importance. **(D)** SHAP beeswarm plot depicting SHAP value distributions across all samples, with color denoting original feature values. **(E)** SHAP waterfall plot demonstrating how the prediction for a representative patient progressed from the base value (E[f(x)]) to the final output (f(x)). **(F)** SHAP force plot visualizing the positive and negative contributions of predictors for the same sample.

The interpretability of the GBM model was evaluated through SHAP analysis. The bar chart revealed that, among lifestyle factors, processed meat intake was the most influential variable. Among the baseline indicators, ALP, NE, and TB emerged as critical predictors. Regarding insulin resistance indices, TyG-BMI and CTI exhibited the highest predictive value (**Figure 4**). The beeswarm plot demonstrated that higher processed meat intake was associated with an elevated risk of developing cancer, whereas higher qualifications and income were correlated with a lower risk of incident cancer (**Figure 4**). Waterfall and force plot analyses confirmed that TyG_bmi made the highest positive contribution to individual predictions (**Figure 4**). To maximize practical utility, we deployed the final model as an interactive online calculator. By inputting routine clinical parameters, healthcare providers can instantly generate a personalized risk probability for patients with CKM syndrome 0-3. Evaluating this score against a designated threshold helps efficiently pinpoint high-risk individuals of cancer, thereby enabling early and targeted interventions (https://ckmstudy.shinyapps.io/makeweb/).

### 3.3 Explanatory analysis via non-linear and mediation models

In the association interpretation phase, we conducted RCS and mediation analyses on the eight continuous variables (DBP, TBIL, ALT, ALP, NE, CTI, TyG-BMI, and Potassium intake) selected by the LASSO, Boruta, and multivariable logistic regression methods. This was performed to further explore their potential mediation pathways with in the risk of incident cancer among patients with CKM syndrome stages 3-4.

#### 3.3.1 Non-linear dose-response associations of the eight core continuous variables

**Figure 5:**
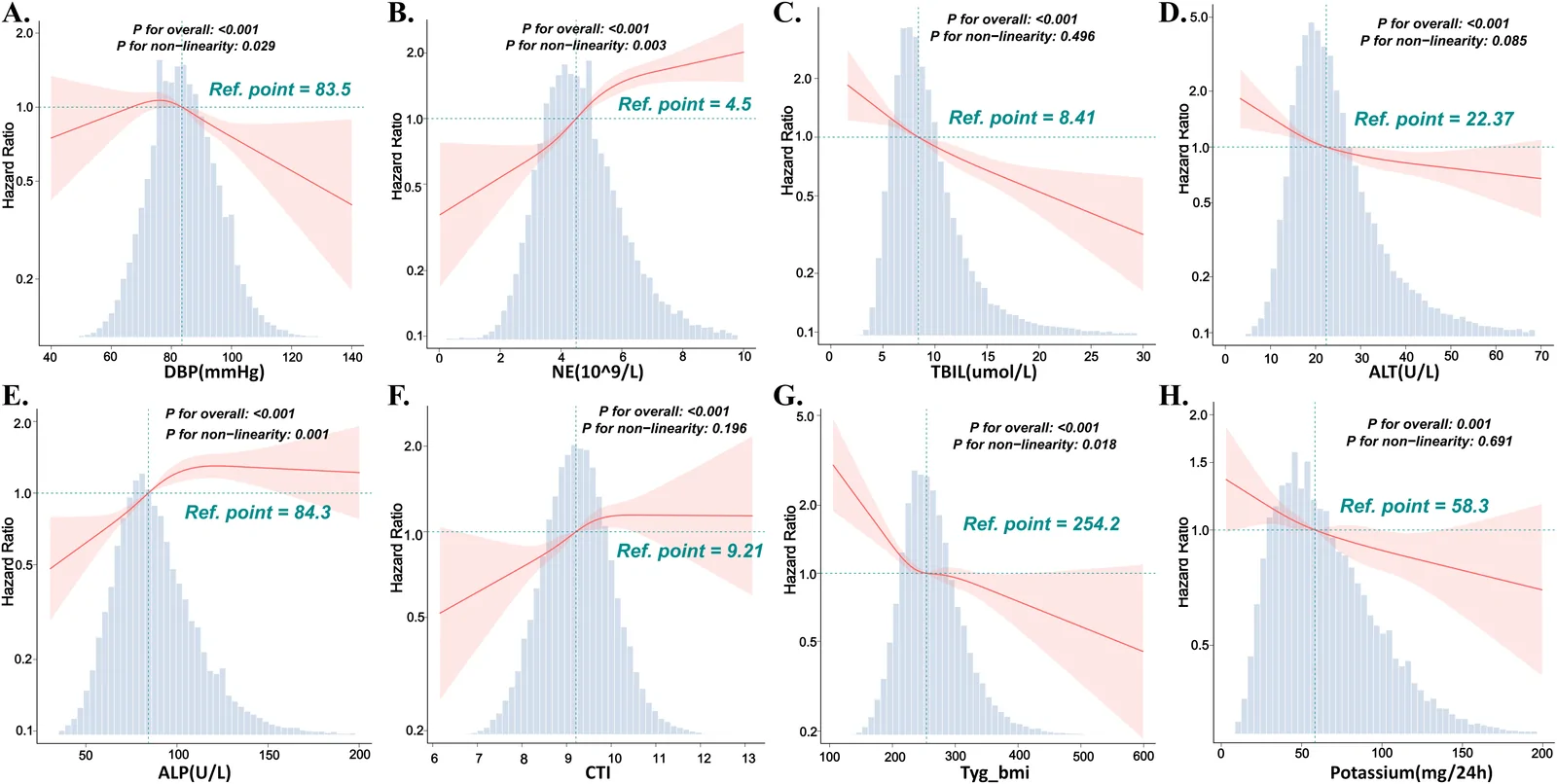
Restricted cubic spline curves for the association between 8 clinical variables and cancer risk in patients with advanced CKM syndrome. **(A)** DBP, **(B)** NE, **(C)** TBIL, **(D)** ALT, **(E)** ALP, **(F)** CTI, **(G)** Tyg-BMI, **(H)** Potassium intake.

To clarify the dose-response relationships between these clinical parameters and the risk of incident cancer, an RCS analysis was performed on the population with CKM syndrome stages 3-4. This analysis focused on the eight continuous variables identified through the multivariable feature selection: DBP, TBIL, ALT, ALP, NE, CTI, TyG-bmi, and potassium intake. The RCS curves revealed that DBP (*P* for overall < 0.001, *P*_non-linearity_ = 0.029), NE (*P* for overall < 0.001, *P*_non-linearity_ = 0.003), ALP (*P* for overall < 0.001, *P*_non-linearity_ = 0.001), and TyG-BMI (*P* for overall < 0.001, *P*_non-linearity_ = 0.018) exhibited significant non-linear associations with cancer risk. In contrast, TBIL (*P* for overall < 0.001, *P*_non-linearity_ = 0.496), ALT (P for overall < 0.001, *P*_non-linearity_ = 0.085), CTI (*P* for overall < 0.001, *P*_non-linearity_ = 0.196), and potassium intake (*P* for overall < 0.001, *P*_non-linearity_ = 0.691) all demonstrated approximately linear relationships (**Figure 5**).

#### 3.3.2 Mediation analysis of the eight core continuous variable

**Figure 6:**
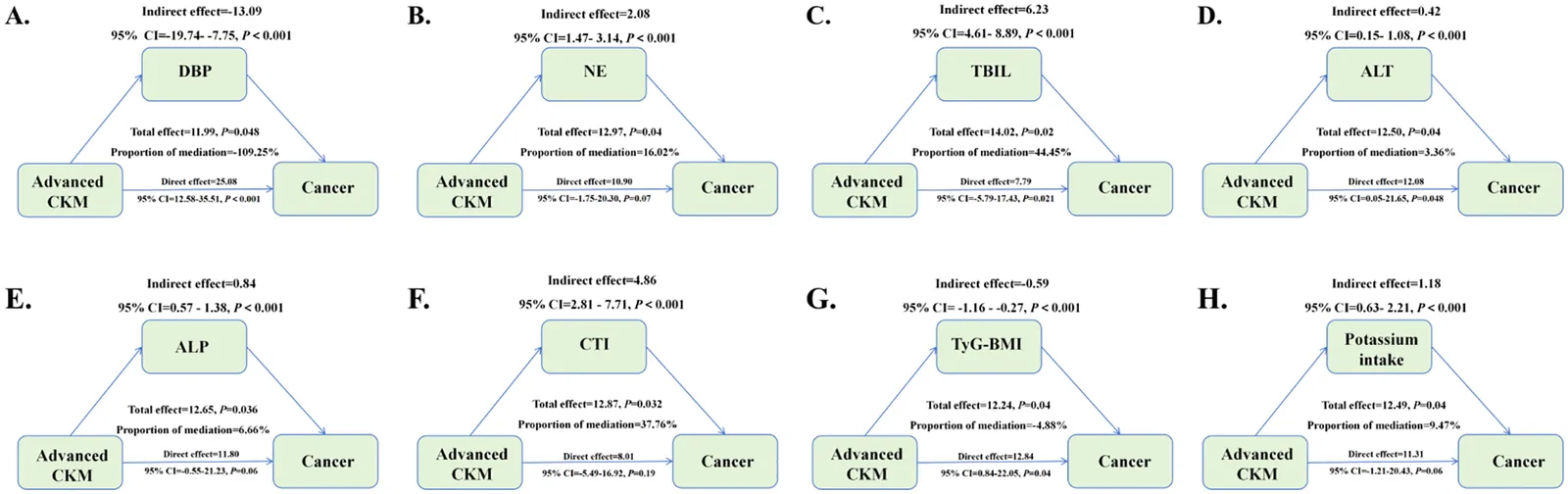
Mediation analysis of 8 clinical variables in the association between advanced CKM syndrome and cancer risk. **(A)** DBP, **(B)** NE, **(C)** TBIL, **(D)** ALT, **(E)** ALP, **(F)** CTI, **(G)** Tyg-BMI, **(H)** Potassium intake.

Multivariable mediation analyses further quantified the statistical contributions of the eight continuous predictors in explaining the association between advanced CKM syndrome and incident cancer (**Figure 6**). All eight variables were significant (P<0.001). Notably, DBP demonstrated a prominent inconsistent mediation effect, characterized by an indirect effect of -13.09 ((95% Cl:-19.74to-7.75, P <0.001), with a statistical suppression proportion of -109.25%. Conversely, TBIL exhibited the strongest consistent positive mediation pathway, with an indirect effect of 6.23 (95% Cl:4.61 to 8.89, P <0.001), accounting for 44.45% of the total statistical association. The remaining indicators, including CTI (37.76%), neutrophil (16.02%), and potassium intake (9.47%), also demonstrated significant positive indirect pathways.

## 4 Discussion

Based on a large-scale prospective cohort of nearly 400,000 individuals from the UK Biobank, this study systematically investigated the dose-response associations and joint associations of CKM syndrome stages and genetic susceptibility on incident cancer risk. Furthermore, an individualized machine learning-based cancer risk prediction model was constructed for the advanced CKM population. The main findings are threefold: (1) advanced CKM stages demonstrate a significant, dose-dependent elevation in cancer risk; (2) a positive interaction exists between genetic risk and CKM syndrome, yielding the highest risk in those with both high genetic susceptibility and advanced CKM; and (3) the GBM model demonstrated robust performance for individualized prediction, maximizing its practical utility through an online risk calculator and leveraging SHAP analysis to highlight major lifestyle and metabolic determinants, while identifying non-linear associations in specific biomarkers and highlighting TBIL as the primary positive mediator.

### Cumulative burden of CKM syndrome and shared pathophysiological mechanisms

This study is the first to confirm an independent association between the 2023 AHA unified CKM staging criteria (1, 19) and cancer risk in a large prospective cohort. Previous studies evaluating cardiometabolic health and cancer largely focused on single components—such as isolated obesity, diabetes, or hypertension (20, 21)—yielding inconsistent conclusions and lacking integrated multi-system assessments (20). Using the comprehensive 0–4 staging system, our results demonstrate that after adjusting for confounders, advanced CKM significantly elevated cancer risk by 2.17 times. This suggests that increased cancer risk stems from the cumulative burden of cardiovascular, renal, and metabolic abnormalities rather than a single metabolic defect. Consequently, advanced CKM syndrome serves as a crucial clinical phenotype for screening high-risk populations.

From a pathophysiological perspective, CKM syndrome and cancer share several core drivers (22): chronic low-grade inflammation (23), insulin resistance (24) oxidative stress (7), and gut microbiota dysbiosis (25). Populations with advanced CKM frequently present with persistently elevated inflammatory cytokines and adipokines, which are linked to DNA damage, impaired immune surveillance, and the promotion of epithelial-mesenchymal transition (EMT), potentially contributing to tumor progression (26). In our model, neutrophil count (NE), alkaline phosphatase (ALP), the C-reactive protein-triglyceride-glucose index (CTI), and TyG_bmi emerged as non-linear core predictors. This finding further supports the premise that systemic inflammation and metabolic dysregulation are potential pathways associated with both CKM syndrome and cancer risk.

### Joint effects of genetic susceptibility and CKM phenotypes

Another vital finding is the joint associations of genetic susceptibility and CKM syndrome. While PRS are effective for stratifying the genetic risk of common cancers (21, 27), their interaction with complex multi-system metabolic phenotypes has remained unclear. This study revealed a significant positive interaction, where individuals with both a high PRS and advanced CKM faced a 3.24-fold increased risk of cancer. This indicates a compounded risk trajectory, where the statistical association with cancer is most pronounced among genetically predisposed individuals with persistent CKM abnormalities. Importantly, this provides a modifiable target for precision prevention: improving CKM health may substantially reduce cancer incidence even in high-genetic-risk populations.

### Machine learning-driven prediction and clinical implications for precision oncology

Targeting the high-risk advanced CKM population, we validated the first machine learning-based prediction model. Machine learning and artificial intelligence are increasingly recognized for their enhanced flexible capacity to integrate complex clinical and multi-omics data and capture potential non-linear relationships (28, 29). Among the tested algorithms, the GBM model achieved the optimal AUC (0.801) in the testing set. Furthermore, by utilizing SHAP analysis (30), we elucidated the specific directional contributions of variables like processed meat intake, TyG-BMI, and TBIL, eliminating the “black-box” limitation of traditional AI and conferring clinical interpretability to the model.

Mediation analysis provided novel explanatory insights, particularly regarding TBIL, which exhibited the strongest positive mediation potential (44.45%). Emerging evidence identifies bilirubin not merely as a marker of liver function, but as an active endocrine and metabolic modulator with potent antioxidant properties that bidirectionally influence cardiometabolic diseases and cancer (31). Conversely, DBP presented a striking negative mediation effect exceeding 100%. Statistically, this represents inconsistent mediation or suppression effect, which occurs when the indirect effect through the mediator counteracts the direct association between the exposure and the outcome. This complex epidemiological phenomenon warrants careful interpretation. Biologically, it may point to a counterbalancing regulatory response or a state of metabolic exhaustion in patients with advanced CKM, where severe multi-organ dysfunction paradoxically couples with altered hemodynamic profiles (32). Methodologically, however, this extreme proportion cannot be entirely decoupled from residual confounding, statistical noise, or reverse mediation. In a long-term prospective setting such as the UK Biobank, individuals with advanced CKM and severely elevated blood pressure face competing risks, such as fatal cardiovascular events, which may selectively remove the highest-risk patients prior to cancer diagnosis. Consequently, the observed association of DBP likely represents a statistical suppression artifact driven by competing survival risks, rather than a genuine biological protection.

### Clinical implications for precision oncology and CKM management

While Cox regression confirms that components like obesity, diabetes, and advanced CKM stages are independent risk factors, it cannot synthesize these dynamic, multi-system parameters into an individualized, absolute probability score at the point of care. Our proposed model effectively translates these findings into a concrete, patient-specific clinical paradigm focused on three primary dimensions: precision stratification, mechanistic tracing, and targeted intervention.

First, regarding precision screening and stratification, the interactive web-based risk calculator (https://ckmstudy.shinyapps.io/makeweb/) shifts clinical workflow from empirical risk awareness to quantified decision-making. CKM patients classified as high-risk by the model can be fast-tracked into intensified or accelerated cancer screening protocols—such as advancing the initiation age or frequency of screening gastroscopies and colonoscopies, or prioritizing high-risk individuals for low-dose spiral computed tomography (LDCT) screening.

Second, our framework dismantles the conventional limitation of machine learning via SHAP interpretability analysis, enabling clinicians to trace the exact phenotypic features associated with a specific patient’s risk.

Third, this visualizable insight informs targeted prevention and management decision-making. For instance, rather than prescribing generalized lifestyle advice, if a patient’s elevated cancer risk is driven predominantly by insulin resistance indices (such as TyG-BMI or CTI) or systemic inflammation (such as neutrophil count), clinicians can implement tailored pharmacological or metabolic interventions. This may include the early, optimized initiation of sodium-glucose cotransporter-2 (SGLT2) inhibitors or glucagon-like peptide-1 (GLP-1) receptor agonists alongside structured nutritional therapy. Consequently, this model serves as a practical clinical bridge, managing advanced CKM syndrome while simultaneously intercepting malignant susceptibility.

### Strengths and limitations

Comparing our findings to existing literature, conventional epidemiological risk models rely heavily on conventional factors like smoking history and age (33). By focusing on the CKM subpopulation and incorporating dynamic biomarkers (inflammation, liver function, and metabolic indices), our GBM model demonstrated promising predictive performance and potential clinical utility for risk stratification.

This study has several limitations.

This study has several limitations. First, the UK Biobank predominantly consists of middle-aged, White individuals, requiring caution when extrapolating our findings to more diverse demographic populations.

Second, CKM staging was assessed exclusively at baseline. Although CKM syndrome is a dynamic continuum and participants may have progressed over the 13.7-year follow-up, time-dependent modeling was precluded because comprehensive staging variables were systematically measured only at initial recruitment. Subsequent repeat assessment waves in the UK Biobank covered only a small, selective fraction of the population, rendering a full-scale time-updated analysis unfeasible for our 12-level joint stratification and machine learning workflows. Consequently, baseline-only assessment may introduce non-differential exposure misclassification. In prospective cohorts, such uncaptured progression typically attenuates effect sizes and biases hazard ratios toward the null, suggesting our reported associations are conservative estimates that may underestimate the true long-term impact of advanced CKM on cancer risk.

Third, our study defined the primary outcome as overall cancer incidence. In this study, defining a combined cancer outcome substantially maximized statistical power, which was methodologically essential to support our multi-variable machine learning training, non-linear splines, and high-order gene-phenotype interaction analyses. However, such an approach inevitably limits the biological interpretation for specific cancer types. Malignant tumors are highly heterogeneous, and different cancer types possess distinct etiologies, metabolic sensibilities, and genetic architectures. While CKM syndrome-induced inflammation, hyperinsulinemia, and oxidative stress promote a generalized pro-tumor environment, these effects may vary significantly by cancer type. Consequently, our findings should be interpreted as a generalized systemic evaluation of malignant susceptibility in the context of CKM syndrome, and future well-powered prospective cohorts are warranted to dissect these relationships within site-specific cancer classifications.

Additionally, a notable limitation is that our optimized model was not subjected to direct, head-to-head empirical comparisons against current tissue-specific clinical screening guidelines or established conventional risk scores in active practice. Therefore, our results should be interpreted as an incremental exploration for risk stratification rather than a definitive superior alternative to current clinical standard tools, which remains an important direction for future validation studies.

## Data Availability

The original contributions presented in the study are included in the article/**Supplementary Material**, further inquiries can be directed to the corresponding author.

## Glossary

CKM: Cardiovascular-kidney-metabolic
TyG: Triglycteride-glucose body mass index
TyG-WHtR: TyG-waist-to-hip ratio
TyG-WC: TyG-waist circumference
SIRI: Systemic Immune-Inflammation Index
NLR: Neutrophil-to-Lymphocyte Ratio
dNLR: Derived Neutrophil-to-Lymphocyte Ratio
PLR: Platelet-to-Lymphocyte Ratio
MLR: Monocyte-to-Lymphocyte Ratio
NMLR: Neutrophil-to-Monocyte Ratio
NPR: Neutrophil to Prealbumin Ratio
CLR: C-reactive protein to Lymphocyte Ratio
PIV: Pan-Immune Inflammation Value
IBI: Inflammatory Burden Index
AISI: Aggregate index of systemic inflammation
LAP: Lipid Accumulation Product
VAI: Visceral Adiposity Index
CVAI: Chinese Visceral Adiposity Index
CTI: C-reactive protein-triglyceride glucose index
eGDR: Estimated Glucose Disposal Rate
HDL-C: High Density Lipoprotein Cholesterol
TyG-BMI: TyG-body mass index
BG: Blood Glucose
HbA1c: Glycated hemoglobin A1c
DBP: Diastolic blood pressure
SBP: Systolic blood pressure
HDL: High Density Lipoprotein
LDL: Low Density Lipoprotein
Hb: Hemoglobin
RDW: Red blood cell distribution width
LYM: Lymphocyte count
NE: Neutrophil count
ALa: Alanine
AST: Aspartate aminotransferase
DBIL: Direct bilirubin
TBIL: Total bilirubin
ALP: Alkaline phosphatase
TP: Total protein
ALB: Albumin
UA: Uric acid
eGFR: estimated glomerular filtration rate
PMI: Processed milk intake
MTU: Milk type used
ORALIH: Own or rent accommodation lived in household
HR: hazard ratios
CI: confidence interval
ML: Machine learning
GBM: Gradient Boosting Machine
SVM: Support Vector Machine
Xgboost: eXtreme Gradient Boosting
Adaboost: Adaptive Boosting
LightGBM: Light Gradient Boosting Machine
CatBoost: Categorical Boosting
SHAP: Shapley Additive Explanations
PRS: polygenic risk scores
EUR: Euro
ROC: Receiver operating characteristic
DCA: Decision curve analysis
