# Correction: Cardiovascular-kidney-metabolic health, genetic susceptibility, and incident cancer risk: a prospective UK biobank cohort and machine learning study

**DOI:** 10.3389/fendo.2026.2006958

**Published:** 2026-10-06

**Authors:** Xue He, Junqiao An, Chunjia Yan, Yutong Zhou, Ziyan Wu, Mengyao Xu, Qingyong He

**Affiliations:** 1 Graduate School, Beijing University of Chinese Medicine, Beijing, China; 2 Guang’anmen Hospital, China Academy of Chinese Medical Sciences, Beijing, China

**Keywords:** cancer, cardiovascular-kidney-metabolic syndrome, machine learning, prospective observational study, UK biobank

The funders “Young Beijing Scholar Program (No. 075), the National Natural Science Foundation of China (No. 82575044) and the Special Program for Cultivating Outstanding Young Scientific and Technological Talents of China Academy of Chinese Medical Sciences (No. CI2023D004)” to “Qing Yong He” were erroneously omitted.

The original version of this article has been updated.

