# Supplementary material for: Cardiovascular-kidney-metabolic health, genetic susceptibility, and incident cancer risk: a prospective UK biobank cohort and machine learning study
Source: Front Endocrinol (Lausanne). 2026 Aug 3;17:1884752. doi: 10.3389/fendo.2026.1884752 (PMC13477978; doi:10.3389/fendo.2026.1884752)
Supplement: Supplementary file 1 [file SupplementaryFile1.docx]

**Supplementary materials**

[Table S1. Definition of CKM syndrome.	2](#_Toc6429)

[Table S2. Ascertainment of disease in the UK Biobank study.	3](#_Toc17405)

[Table S3. Field IDs used for the study.	4](#_Toc15170)

[Table S4. Candidate variables and calculation methods.	5](#_Toc16178)

[Table S5. The association of stages of CKM syndrome with risk of cancer after excluding cancer cases with follow-up time < 2 years.	8](#_Toc16854)

[Table S6. Baseline characteristics of the study population across training and internal test cohorts.	9](#_Toc27523)

[Table S7. Collinearity Analysis of Selected Variables.	13](#_Toc736)

[Table S8. Identification of Independent Predictors for RBD Based on Multivariate Logistic Regression.	14](#_Toc17414)

[Table S9. Performance of eight machine learning models in the training and internal test sets.	22](#_Toc23949)

[Table S10. Pairwise DeLong Test Z-statistics and P-values for AUC Comparisons Among the Eight Machine Learning Models.	23](#_Toc25085)

[Figure S1. Flowchart for the selection of the study population from the UK Biobank study.	25](#_Toc27090)

[Figure S2. Comulative risk of cancer.	26](#_Toc14937)

[References	27](#_Toc23135)

**Table S1. Definition of CKM syndrome.**

| **Stage** | **Definition** |
| --- | --- |
| **0** | Normal BMI  (Asian＜23 kg/m^2^, Other races and ethnicities＜25 kg/m^2^)  Normal waist circumference  (Waist circumference＜80/90 cm in Asian women/men, Waist circumference＜88/102cm in women and men of other races and ethnicities)  And participants who do not meet the standards of other stages |
| **1** | Elevated BMI  (Asian≥23 kg/m^2^, Other races and ethnicities≥25 kg/m^2^)  Elevated waist circumference  (Waist circumference ≥ 80/90cm in Asian women/men, Waist circumference ≥88/102cm in women and men of other races and ethnicities)  Or prediabetes  (HbA1c between 5.7% and 6.5% or Fasting blood glucose between 100mg/dL and 126 mg/dL) |
| **2** | Participants with metabolic risk factors or those with moderate-to-high risk CKD according to KDIGO criteria  Metabolic risk factors that meet the criteria include: Elevated fasting serum triglycerides(≥135 mg/dL), Hypertension, Diabetes or Metabolic Syndrome (The following≥3 items: Elevated waist circumference, Low HDL levels(<40/50 mg/dL in men and women), Fasting Serum Triglycerides≥150 mg/dL, Elevated blood pressure(SBP≥130, DBP≥80 mmHg, and/or using antihypertensive drugs  ) or prediabetes)  Moderate-to-high risk CKD  Determine CKD stage using eGFR and ACR, the calculation method of eGFR adopts New Creatinine- and Cystatin C-Based Equations to Estimate GFR without Race^[1]^.  Moderate risk: eGFR: 45-59ml/min/1.73m²; and  ACR: 30-299mg/g or 3-29mg/mmol  high risk: eGFR: 30-44ml/min/1.73m²; and  ACR≥30mg/g or≥3mg/mmol |
| **3** | Extremely high-risk CKD or estimate 10-year risk of cardiovascular disease  Extremely high-risk chronic kidney disease  Extremely high-risk: eGFR <30 mL/min/1.73m² or  ACR ≥300 mg/g  Estimate 10-year risk of cardiovascular disease  10-year risk of cardiovascular disease use SCORE2^[2]^ for estimation high risk defined as a 10-year cardiovascular disease risk of ≥10% (Based on the recommended threshold) |
| **4** | Confirmed cardiovascular disease  (Coronary Heart Disease, Angina pectoris, Heart attack, HF, atrial fibrillation, Peripheral artery disease and stroke) |

**Table S2. Ascertainment of disease in the UK Biobank study.**

| **Disease** | **ICD-10** |
| --- | --- |
| Cancer | C00-C97 |
| Hypertension | I10, I11, I12, I13, I15 |
| Diabetes | E10 to E14 |
| Coronary heart disease | I20 to I25 |
| Atrial fibrillation | I48 |
| Stroke | I60, I61, I62, I63 |
| Heart failure | I50 |
| Peripheral artery disease | I70, I71, I72, I73, I74, I77, I78, I79 |

**Table S3. Field IDs used for the study.**

| **Field ID** | **Variables** |
| --- | --- |
| 31 | Sex |
| 21022 | Age at recruitment |
| 21000 | Ethnic background |
| 22189 | Townsend deprivation index at recruitment |
| 6138 | Qualification |
| 738 | Average total household income before tax |
| 21001 | Body mass index (BMI) |
| 48 | Waist circumference |
| 20116 | Smoking status |
| 20117 | Alcohol drinker status |
| 22040 | Summed MET minutes per week for all activity |
| 20107 | Illnesses of father |
| 20110 | Illnesses of mother |
| 20111 | Illnesses of siblings |
| 30750 | Glycated haemoglobin (HbA1c) |
| 30870 | Triglycerides |
| 30690 | Cholesterol |
| 30760 | HDL Cholesterol |
| 23404 | LDL Cholesterol |
| 30700 | Creatinine |
| 6177 | Medication for cholesterol, blood pressure or diabetes |
| 20003 | Treatment/medication code |
| 30740 | Blood glucose |
| 4079 | Diastolic blood pressure, automated reading |
| 4080 | Systolic blood pressure, automated reading |
| 30500 | Microalbumin in urine |
| 30720 | Cystatin C |

**Table S4. Summary of SNPs and statistical parameters used for polygenic risk score construction. SNPs, single nucleotide polymorphisms.**

| **riskAllele** | **pValue** | **Risk**  **Frequency** | **orValue** | **ci** | **mappedGenes** | **accessionId** | **locations** |
| --- | --- | --- | --- | --- | --- | --- | --- |
| rs10015491-C | 1E-10 | 0.054 | 1.23 | (1.16, 1.31) | TRIM48,OR4A6P | GCST011108 | 11:54880568 |
| rs13361707-C | 1E-10 | 0.059 | 0.8 | (0.74, 0.86) | STK10 | GCST011108 | 5:172151880 |
| rs6676150-C | 1E-10 | 0.062 | 1.21 | (1.14, 1.29) | NRXN1-DT | GCST011108 | 2:51959960 |
| rs3999544-C | 1E-10 | 0.07 | 1.26 | (1.16, 1.38) | SUCLG2 | GCST003100 | 3:67392218 |
| rs10993994-T | 1E-10 | 0.08 | 1.83 | (1.51, 2.22) | ELOVL5 | GCST003230 | 6:53312880 |
| rs4713266-C | 1E-10 | 0.09 | 1.56 | (1.3, 1.87) | LINC01919,LINC01917 | GCST003230 | 18:53580709 |
| rs61751053-T | 1E-10 | 0.196 | 0.88 | (0.84, 0.92) | HDAC9 | GCST011108 | 7:18931900 |
| rs35322910-T | 1E-10 | 0.299 | 0.89 | (0.86, 0.93) | ROBO2P1,CICP17 | GCST011108 | 7:51473441 |
| rs2271920-A | 1E-11 | 0.0532 | 2.5468225 | (1.74, 3.72) | CHCHD3 | GCST90832127 | 7:132843748 |
| rs12913538-A | 1E-11 | 0.0539 | 2.884026 | (1.9, 4.37) | SORCS1 | GCST90832127 | 10:106636450 |
| rs2285803-T | 1E-11 | 0.066 | 2.5855412 | (1.77, 3.78) | TTC6 | GCST90832127 | 14:38008106 |
| rs72797404-C | 1E-11 | 0.067 | 2.7370343 | (1.91, 3.92) | LTBP1 | GCST90832127 | 2:33089897 |
| rs117618124-T | 1E-11 | 0.0705 | 2.3875978 | (1.65, 3.46) | ISX,LINC02885 | GCST90832127 | 22:35019584 |
| rs2248661-A | 1E-11 | 0.074 | 2.5157266 | (1.77, 3.58) | BDNF,CBX3P1 | GCST90832127 | 11:27758984 |
| rs11892031-A | 1E-11 | 0.0742 | 2.4189079 | (1.71, 3.43) | DYRK3-AS1,EIF2D | GCST90832127 | 1:206612973 |
| rs7103831-G | 1E-11 | 0.081 | 2.2326105 | (1.64, 3.04) | ARHGAP33 | GCST90832127 | 19:35777419 |
| rs7820838-T | 1E-11 | 0.0862 | 2.3811278 | (1.67, 3.4) | SMIM19,CHRNB3 | GCST90832127 | 8:42563297 |
| rs35951924-A | 1E-11 | 0.0916 | 2.1852646 | (1.57, 3.04) | RAB12 | GCST90832127 | 18:8621422 |
| rs6791479-T | 1E-11 | 0.0974 | 2.3546999 | (1.68, 3.29) | AKAIN1 | GCST90832127 | 18:5145390 |
| rs798766-T | 1E-11 | 0.098 | 2.2789135 | (1.68, 3.08) | DCHS2,SFRP2 | GCST90832127 | 4:153970109 |
| rs6700380-T | 1E-11 | 0.0986 | 2.0658839 | (1.53, 2.8) | AREG,BTC | GCST90832127 | 4:74704309 |
| rs8102137-C | 1E-11 | 0.0996 | 2.330054 | (1.7, 3.19) | LHFPL2 | GCST90832127 | 5:78616101 |
| rs2735940-A | 1E-11 | 0.111 | 1.48 | (1.3, 1.69) | LINC01500 | GCST90102458 | 14:58669547 |
| rs1014971-T | 1E-11 | 0.1156 | 2.1519291 | (1.61, 2.88) | ZAR1L | GCST90832127 | 13:32305445 |
| rs2351179-C | 1E-11 | 0.122 | 0.4 | (0.3, 0.6) | KCNQ4 | GCST90179123 | 1:40819620 |
| rs56069439-A | 1E-11 | 0.1237 | 1.988223 | (1.49, 2.65) | RASGRP4 | GCST90832127 | 19:38411020 |
| rs4711748-T | 1E-11 | 0.128 | 2.3 | (1.6, 3.3) | DLGAP2 | GCST90179123 | 8:877824 |
| rs17715065-C | 1E-11 | 0.1308 | 2.0318766 | (1.5, 2.75) | HAO2 | GCST90832127 | 1:119385564 |
| rs11388275-A | 1E-11 | 0.135 | 0.4 | (0.3, 0.6) | SP140,SP110 | GCST90179123 | 2:230218456 |
| rs6815814-A | 1E-11 | 0.1352 | 1.9276003 | (1.46, 2.55) | SGO1-AS1 | GCST90832127 | 3:20329077 |
| rs149866169-A | 1E-11 | 0.141 | 1.9793217 | (1.5, 2.61) | GCGR,SLC25A10 | GCST90832127 | 17:81726106 |
| rs73094911-C | 1E-11 | 0.166 | 1.8226407 | (1.42, 2.34) | DYSF,RPS20P10 | GCST90832127 | 2:71751751 |
| rs5924562-C | 1E-11 | 0.174 | 0.5 | (0.3, 0.6) | PTCHD3,TRIAP1P1 | GCST90179123 | 10:27395731 |
| rs7959150-A | 1E-11 | 0.1972 | 1.9250715 | (1.49, 2.48) | LINC01951 | GCST90832127 | 5:174952467 |
| rs7190458-A | 1E-11 | 0.2265 | 1.8487523 | (1.44, 2.37) | OSBPL10 | GCST90832127 | 3:31849233 |
| rs1294247-T | 1E-11 | 0.228 | 0.5 | (0.4, 0.7) | BMP3 | GCST90179123 | 4:81056536 |
| rs55668940-A | 1E-11 | 0.2917 | 1.7961316 | (1.41, 2.29) | VLDLR-AS1 | GCST90832127 | 9:2415379 |
| rs8030672-A | 1E-11 | 0.31 | 1.49 | (1.27, 1.74) | TENM3-AS1 | GCST002596 | 4:181892145 |
| rs365222-A | 1E-11 | 0.353 | 1.8 | (1.4, 2.3) | HLA-DQA1,HLA-DRB1 | GCST90179123 | 6:32619017 |
| rs2965183-A | 1E-11 | 0.4014 | 1.6189251 | (1.32, 1.99) | PCNT | GCST90832127 | 21:46363117 |
| rs720475-G | 1E-11 | 0.411 | 1.6835899 | (1.35, 2.1) | BLOC1S5-TXNDC5 | GCST90832127 | 6:7917701 |
| rs116461399-C | 1E-11 | 0.427 | 1.7483382 | (1.4, 2.19) | RFC3P1,RPL36AP17 | GCST90832127 | 3:36040639 |
| rs7712641-C | 1E-11 | 0.428 | 1.6862472 | (1.37, 2.07) | RHOB,Y_RNA | GCST90832127 | 2:20480097 |
| rs7040024-A | 1E-11 | 0.446 | 0.5222386 | (0.42, 0.66) | ERI1,RPL10P19 | GCST90832127 | 8:8976730 |
| rs514192-A | 1E-11 | 0.45 | 0.58676857 | (0.47, 0.74) | PVT1 | GCST90832127 | 8:128072767 |
| rs114828403-C | 1E-11 | 0.46 | 1.6276958 | (1.33, 1.99) | PLB1 | GCST90832127 | 2:28480602 |
| rs11067228-A | 1E-11 | 0.476 | 0.5 | (0.4, 0.7) | GRIK2,R3HDM2P2 | GCST90179123 | 6:102762890 |
| rs78540526-T | 1E-11 | 0.9 | 1.19 | (1.11, 1.28) | SMLR1,Y_RNA | GCST002596 | 6:130604622 |
| rs10852606-C | 1E-11 | 0.46-0.52 | 1.11 | (1.06, 1.15) | TNS3,SRSF8CP | GCST002415 | 7:47199109 |
| rs13328298-G | 1E-11 | 0.47-0.54 | 1.12 | (1.08, 1.18) | ACOXL | GCST002415 | 2:110970905 |
| rs16891982-G | 1E-112 | 0.51-0.56 | 1.11 | (1.07, 1.16) | ST8SIA2,NPM1P5 | GCST002415 | 15:92316651 |
| rs6983267-G | 1E-113 | 0.41-0.49 | 1.11 | (1.06, 1.16) | LINC01798 | GCST002415 | 2:66738934 |
| rs60681470-A | 1E-119 | 0.51-0.58 | 1.14 | (1.08, 1.2) | C3orf85 | GCST002415 | 3:109132637 |
| rs6059655-G | 1E-12 | 0.0597 | 1.14 | (1.1, 1.18) | SLC6A18 | GCST007856 | 5:1240089 |
| rs72797404-T | 1E-12 | 0.0829 | 1.2 | (1.17, 1.24) | EIF3H,LINC00536 | GCST007856 | 8:116618444 |
| rs4236709-G | 1E-12 | 0.0983 | 1.1 | (1.07, 1.14) | VTI1A | GCST007856 | 10:112528860 |
| rs6658977-T | 1E-12 | 0.139 | 1.1627907 | (1.1, 1.23) | TMTC1 | GCST003209 | 12:29680396 |
| rs1229984-C | 1E-12 | 0.1523 | 1.09 | (1.07, 1.12) | RN7SL547P,SRSF10P2 | GCST007856 | 20:7760329 |
| rs72834823-T | 1E-12 | 0.1529 | 1.07 | (1.04, 1.09) | RIPOR3 | GCST007856 | 20:50639748 |
| rs2277283-C | 1E-12 | 0.2178 | 1.06 | (1.03, 1.08) | TCF7L2 | GCST007856 | 10:112962862 |
| rs4795519-C | 1E-12 | 0.2626 | 1.08 | (1.06, 1.1) | NKX2-3,SLC25A28 | GCST007856 | 10:99591947 |
| rs187809440-T | 1E-12 | 0.264 | 1.0869565 | (1.05, 1.12) | GMCL1 | GCST003208 | 2:69832692 |
| rs116260619-C | 1E-12 | 0.267 | 1.06 | (1.04, 1.08) | PLEKHG6,ATP5MFP5 | GCST007856 | 12:6297738 |
| rs228867-A | 1E-12 | 0.277 | 1.0869565 | (1.05, 1.12) | LITAF | GCST003208 | 16:11626210 |
| rs4236709-G | 1E-12 | 0.288 | 1.1 | (1.08, 1.12) | RNU1-150P,TTC33 | GCST007856 | 5:40279974 |
| rs992530-A | 1E-12 | 0.352 | 1.0752687 | (1.04, 1.11) | MGAT5B | GCST003208 | 17:76934284 |
| rs56868629-A | 1E-12 | 0.3564 | 1.06 | (1.04, 1.08) | KRT18P56,LINC00604 | GCST007856 | 5:40102341 |
| rs10845938-G | 1E-12 | 0.363 | 1.1111112 | (1.06, 1.16) | MIR6130,MAPK6P2 | GCST003209 | 21:23048180 |
| rs1976052-T | 1E-12 | 0.3761 | 1.15 | (1.13, 1.17) | POU5F1B,PCAT1,CASC8 | GCST007856 | 8:127402647 |
| rs4784227-T | 1E-12 | 0.3823 | 1.06 | (1.04, 1.08) | RNU7-147P,PLCL1 | GCST007856 | 2:198747683 |
| rs2494938-A | 1E-12 | 0.4016 | 1.06 | (1.04, 1.08) | LINC01500 | GCST007856 | 14:58741719 |
| rs142727307-T | 1E-12 | 0.447 | 1.0752687 | (1.05, 1.11) | LINC02719,GUCY1A2 | GCST003208 | 11:106436144 |
| rs1160267-G | 1E-12 | 0.461 | 1.0752687 | (1.04, 1.11) | CDKN2B-AS1 | GCST003208 | 9:22121350 |
| rs2793110-C | 1E-12 | 0.4976 | 1.05 | (1.03, 1.07) | LINC00917,LINC01081 | GCST007856 | 16:86305709 |
| rs9693444-A | 1E-12 | 0.513 | 1.12 | (1.07, 1.16) | SCAF8 | GCST003209 | 6:154800125 |
| rs17694493-G | 1E-12 | 0.52 | 1.1 | (1.06, 1.13) | ATXN2,SH2B3 | GCST003208 | 12:111446804 |
| rs2046210-A | 1E-12 | 0.55 | 1.11 | (1.06, 1.16) | RETREG3 | GCST003209 | 17:42603793 |
| rs1420285-C | 1E-12 | 0.55 | 1.1494253 | (1.09, 1.22) | LRRC4C | GCST003229 | 11:41047350 |
| rs13263376-A | 1E-12 | 0.5635 | 1.06 | (1.04, 1.08) | LINC01271,RN7SL636P | GCST007856 | 20:50367160 |
| rs6597981-G | 1E-12 | 0.649 | 1.12 | (1.08, 1.17) | FAM182A | GCST003209 | 20:25995038 |
| rs8045257-A | 1E-12 | 0.69 | 1.08 | (1.05, 1.12) | NPAS3 | GCST003208 | 14:33820232 |
| rs9266329-A | 1E-12 | 0.6981 | 1.05 | (1.03, 1.07) | RNA5SP299,CHCHD3P1 | GCST007856 | 10:8621912 |
| rs214831-G | 1E-12 | 0.703 | 1.09 | (1.07, 1.12) | RNA5SP299,LINC02676 | GCST007856 | 10:8697617 |
| rs411482-C | 1E-12 | 0.7095 | 1.07 | (1.04, 1.09) | PLEKHG6 | GCST007856 | 12:6312008 |
| rs11196172-A | 1E-12 | 0.725 | 1.11 | (1.06, 1.17) | GDPD5,SERPINH1 | GCST003209 | 11:75537412 |
| rs7621631-A | 1E-12 | 0.762 | 1.05 | (1.03, 1.08) | NKX2-3,SLC25A28 | GCST007856 | 10:99555409 |
| rs9745545-C | 1E-12 | 0.767 | 1.12 | (1.07, 1.18) | LIMA1 | GCST003209 | 12:50185615 |
| rs7741164-A | 1E-12 | 0.7712 | 1.07 | (1.04, 1.1) | LINC00536,EIF3H | GCST007856 | 8:116620726 |
| rs16901979-A | 1E-12 | 0.773 | 1.1 | (1.06, 1.14) | ECT2L | GCST003208 | 6:138801103 |
| rs7707921-A | 1E-12 | 0.782 | 1.15 | (1.09, 1.22) | CPSF6P1,ZNF429 | GCST003209 | 19:21569009 |
| rs28557075-A | 1E-12 | 0.8102 | 1.05 | (1.03, 1.08) | MAP2K5 | GCST007856 | 15:67768051 |
| rs71559437-A | 1E-12 | 0.862 | 1.17 | (1.11, 1.25) | RNGTT | GCST003209 | 6:88856018 |
| rs17486278-C | 1E-12 | 0.8624 | 1.06 | (1.04, 1.09) | CHRDL2 | GCST007856 | 11:74698032 |
| rs10758203-A | 1E-12 | 0.8732 | 1.09 | (1.07, 1.12) | NXN | GCST007856 | 17:911003 |
| rs139244745-G | 1E-12 | 0.8755 | 1.06 | (1.04, 1.09) | HLA-B,LINC02571 | GCST007856 | 6:31347735 |
| rs7568458-T | 1E-12 | 0.876 | 1.13 | (1.08, 1.18) | TET2,RNU6-351P | GCST003208 | 4:105143597 |
| rs2450115-T | 1E-12 | 0.891 | 1.18 | (1.1, 1.26) | COX6B1P1,RNU6-650P | GCST003209 | 7:149043401 |
| rs143383-A | 1E-12 | 0.898 | 1.15 | (1.09, 1.21) | LSAMP | GCST003208 | 3:115931601 |
| rs2981578-C | 1E-12 | 0.912 | 1.21 | (1.13, 1.31) | MALRD1,AIFM1P1 | GCST003209 | 10:19031157 |
| rs72801582-C | 1E-12 | 0.929 | 1.16 | (1.09, 1.24) | HOXB1,HOXB2 | GCST003208 | 17:48536888 |
| rs11196172-A | 1E-12 | 0.934 | 1.22 | (1.12, 1.33) | CSRNP3,GALNT3 | GCST003209 | 2:165696408 |
| rs3129859-G | 1E-12 | 0.944 | 1.27 | (1.15, 1.39) | CLEC3A | GCST003209 | 16:78042662 |

**Table S5. Candidate variables and calculation methods.**

| **Variables** | **Field ID** | **Calculation methods** |
| --- | --- | --- |
| Age | 21022 | / |
| Sex | 31 | / |
| Average total household income before tax | 738 | / |
| Qualification | 6138 | / |
| Ethnic | 21000 | / |
| Drinking | 20117 | / |
| Cancer of family history | 20107、20110、20111 | / |
| Skin colour | 1717 | / |
| Milk type used | 1418 | / |
| Processed meat intake | 1349 | / |
| Insomnia | 1200 | / |
| Daytime dozing | 1220 | / |
| Length of mobile phone use | 1110 | / |
| Own or rent accommodation  lived in Household | 680 | / |
| Number of vehicles in household | 728 | / |
| Frequency of friend/family visits | 1031 | / |
| Able to confide | 2110 | / |
| Breastfed as a baby | 1677 | / |
| Irritability | 1940 | / |
| Weight change compared with 1 year ago | 2306 | / |
| TyG | 30740、30870 | TyG=ln[FPG (mmol/L)×TG (mmol/L)÷2] |
| TyG-WHtR | 30740、30870、48、12144 | TyG=ln[FPG (mmol/L)×TG (mmol/L)÷2]  WHtR= Height (cm)÷Waist circumference (cm)  TyG-WHtR=TyG×WHtR |
| TyG-WC | 30740、30870、48 | TyG-WC=TyG=ln[FPG (mmol/L)×TG (mmol/L)÷2]×Waist circumference (cm) |
| TyG-BMI | 30740、30870、21001 | TyG-WC=TyG=ln[FPG (mmol/L)×TG (mmol/L)÷2]×BMI (Kg/m^2^) |
| SIRI | 30200、30120、 |  |
| NIR | 30200、30120 |  |
| dNLR | 30200 |  |
| PLR | 30120、30080 |  |
| MLR | 30120 |  |
| NPR | 30200、30080 |  |
| CLR | 30120 |  |
| LAP | 48、30870 |  |
| VAI | 48、30870、30760、21001 |  |
| CVAI | 48、30870、30760、21001 |  |
| CTI | 30760、30740 |  |
| IBI | 30120、30200 |  |
| NON-HDL-C/HDL-C Ratio | 30760、30690 |  |
| eGDR | 48、30750 | eGDR (mg/kg/min) = 21.158 - (0.09 × Waist circumference(cm)) - (3.407 × Hypertension) - (0.551× HbA1c%) |
| PIV | 30200、30120、30080、30130 |  |
| Townsend | 22189 | / |
| Physical activity | 22040 | / |
| Blood Glucose | 30740 | / |
| HbA1c | 30750 | / |
| Diastolic blood pressure, automated reading | 4079 | / |
| Systolic blood pressure, automated reading | 4080 | / |
| HDL-C | 30760 | / |
| LDL-C | 23404 | / |
| Hb | 30020 | / |
| RDW | 30700 | / |
| LYM | 30120 | / |
| NE | 30140 | / |
| ALT | 30620 | / |
| AST | 30650 | / |
| DBIL | 30660 | / |
| TBIL | 30840 | / |
| ALP | 30610 | / |
| TP | 30860 | / |
| ALB | 30600 | / |
| UA | 30880 | / |
| eGFR | 30700、30720 | / |
| Potassium intake | 26024 | / |

**Table S6. Comparison of baseline characteristics between participants included in the final analysis and those excluded due to incomplete CKM syndrome data.**

|  | No | Yes | p | SMD |
| --- | --- | --- | --- | --- |
| n | 399034 | 75589 |  |  |
| **Sociodemographic factors** |  |  |  |  |
| Age at recruitment (years) | 56.56 (8.09) | 56.45 (8.10) | 0.001 | 0.014 |
| Female, n (%) | 214518 ( 53.8) | 42979 ( 56.9) | <0.001 | 0.062 |
| White ethnicity, n (%) | 352989 ( 88.5) | 67256 ( 89.0) | <0.001 | 0.016 |
| Townsend deprivation index | -1.32 (3.07) | -1.21 (3.19) | <0.001 | 0.035 |
| College or university degree, n (%) | 130153 ( 32.6) | 24916 ( 33.0) | 0.064 | 0.007 |
| Income before tax >52,000 EUR, n (%) | 99986 ( 25.1) | 18124 ( 24.0) | <0.001 | 0.025 |
| **Lifestyle information** |  |  |  |  |
| BMI (kg/m2) | 27.41 (4.75) | 27.54 (5.00) | <0.001 | 0.026 |
| Waist circumference (cm) | 90.39 (13.44) | 90.04 (13.66) | <0.001 | 0.025 |
| Current smoker, n (%) | 41922 ( 10.5) | 8287 ( 11.0) | <0.001 | 0.015 |
| Current drinking, n (%) | 367074 ( 92.0) | 69246 ( 91.6) | <0.001 | 0.014 |
| Physical activity (MET min/week) | 2654.94 (2662.40) | 2627.06 (2632.11) | 0.008 | 0.011 |
| Family history of cancer, n (%) | 175465 ( 44.0) | 33386 ( 44.2) | 0.323 | 0.004 |
| **Laboratory measurements** |  |  |  |  |
| Blood glucose (mmol/L) | 92.38 (22.25) | 92.00 (23.99) | 0.010 | 0.016 |
| HbAlc (mmol/mol) | 36.12 (6.75) | 36.15 (6.82) | 0.335 | 0.004 |
| Total Cholesterol (mmol/L) | 5.69 (1.14) | 5.69 (1.16) | 0.450 | 0.003 |
| Triglycerides (mmol/L) | 1.75 (1.02) | 1.76 (1.03) | 0.012 | 0.010 |
| HDL-C (mmol/L) | 56.04 (14.80) | 55.46 (14.70) | <0.001 | 0.040 |
| LDL-C (mmol/L) | 2.59 (0.74) | 2.60 (0.75) | 0.010 | 0.010 |
| Systolic blood pressure (mmHg) | 137.83 (18.60) | 137.53 (18.83) | 0.001 | 0.017 |
| Diastolic blood pressure (mmHg) | 82.24 (10.12) | 81.91 (10.16) | <0.001 | 0.032 |

**Table S7. The association of stages of CKM syndrome with risk of cancer after excluding cancer cases with follow-up time < 2 years.**

| **Exposure** | **Model 1**  **HR (95% CI)** | ***P*** | **Model 2**  **HR (95% CI)** | ***P*** | **Model 3**  **HR (95% CI)** | ***P*** |
| --- | --- | --- | --- | --- | --- | --- |
| Stage 0 | **Ref** |  | **Ref** |  | **Ref** |  |
| Stage 1 | 1.20 (1.08, 1.34) | 0.001 | 1.06 (0.95, 1.18) | 0.327 | 1.00 (0.90, 1.11) | 0.961 |
| Stage 2 | 1.58 (1.44, 1.75) | <0.001 | 1.30 (1.18, 1.43) | <0.001 | 1.16 (1.05, 1.28) | 0.003 |
| Stage 3-4 | 4.57 (4.11, 5.07) | <0.001 | 2.53 (2.26, 2.83) | <0.001 | 1.37 (1.22, 1.54) | <0.001 |

Note: These models utilized the Cox proportional hazards model. Model 1 was unadjusted. Model 2 was adjusted for age, sex, ethnic. Model 3 was adjusted for age, sex, ethnic, Townsend, cancer of family history, physical activity, drinking and smoking.

**Table S8. Baseline characteristics of the study population across training and internal test cohorts.**

| **Variables** | **Train**  **（n=23031）** | **Test**  **（n=9870）** | ***P*** | **SMD** |
| --- | --- | --- | --- | --- |
| **Sex, n(%)** |  |  | 0.693 | 0.005 |
| Female | 5201 (22.6) | 2192 (22.2) |  |  |
| Male | 17830 (77.4) | 7678 (77.8) |  |  |
| **Age (%)** | 63.27(5.58) | 63.26(5.59%) | 0.356 | 0.011 |
| **Income, n(%)** |  |  | 0.7 | 0.005 |
| Income before tax >52,000 EUR | 2552 (11.1) | 1082 (11.0) |  |  |
| Income before tax ≤52,000 EUR | 20479 (88.9) | 8788 (89.0) |  |  |
| **Qualification, n(%)** |  |  | 0.433 | 0.009 |
| College or university degree | 4883 (21.2) | 2096 (21.2) |  |  |
| Not College or university degree | 18148 (78.8) | 7774 (78.8) |  |  |
| **Ethnic, n(%)** |  |  | 0.202 | 0.015 |
| Other | 21123 (91.7) | 9089 (92.1) |  |  |
| White | 1908 (8.3) | 781 (7.9) |  |  |
| **Drinking, n(%)** |  |  | 0.25 | 0.014 |
| Yes | 20867 (90.6) | 8919 (90.4) |  |  |
| No | 2164 (9.4) | 951 (9.6) |  |  |
| **Cancer of family history, n(%)** |  |  | 0.563 | 0.007 |
| Yes | 11005 (47.8) | 4711 (47.7) |  |  |
| No | 12026 (52.2) | 5159 (52.3) |  |  |
| **Skin colour, n(%)** |  |  | 0.667 | 0.021 |
| Fair | 16842 (73.1) | 7234 (73.3) |  |  |
| Light olive | 3773 (16.4) | 1611 (16.3) |  |  |
| Very fair | 1698 (7.4) | 747 (7.6) |  |  |
| Brown | 718 (3.1) | 278 (2.8) |  |  |
| **Milk type used, n(%)** |  |  | 0.693 | 0.021 |
| Semi-skimmed | 15563 (67.6) | 6728 (68.2) |  |  |
| Skimmed | 4824 (20.9) | 2036 (20.6) |  |  |
| Soya | 791 (3.4) | 304 (3.1) |  |  |
| Full cream | 1853 (8.0) | 802 (8.1) |  |  |
| **Processed meat intake, n(%)** |  |  | 0.436 | 0.023 |
| 2-4 times a week | 5736 (24.9) | 2514 (25.5) |  |  |
| Once a week | 7001 (30.4) | 3016 (30.6) |  |  |
| Less than once a week | 8095 (35.1) | 3383 (34.3) |  |  |
| Never | 1249 (5.4) | 544 (5.5) |  |  |
| 5-6 times a week | 950 (4.1) | 413 (4.2) |  |  |
| **Insomnia, n(%)** |  |  | 0.618 | 0.012 |
| Usually | 7364 (32.0) | 3151 (31.9) |  |  |
| Sometimes | 10388 (45.1) | 4448 (45.1) |  |  |
| Never/rarely | 5279 (22.9) | 2271 (23.0) |  |  |
| **Daytime dozing, n(%)** |  |  | 0.77 | 0.009 |
| Never/rarely | 15471 (67.2) | 6632 (67.2) |  |  |
| Sometimes | 6478 (28.1) | 2767 (28.0) |  |  |
| Often | 1082 (4.7) | 471 (4.8) |  |  |
| **Length of mobile phone use, n(%)** | | | 0.704 | 0.018 |
| Five to eight years | 6051 (26.3) | 2647 (26.8) |  |  |
| Two to four years | 5054 (21.9) | 2159 (21.9) |  |  |
| More than eight years | 7101 (30.8) | 3032 (30.7) |  |  |
| Never used mobile phone at least once per week | 3985 (17.3) | 1734 (17.6) |  |  |
| One year or less | 840 (3.6) | 298 (3.0) |  |  |
| **Own or rent accommodation lived in Household, n(%)** | | | 0.019 | 0.019 |
| Rent-from private landlord or letting agency | 718 (3.1) | 287 (2.9) |  |  |
| Own outright (by you or someone in your household) | 15649 (67.9) | 6668 (67.6) |  |  |
| Own with a mortgage | 4279 (18.6) | 1968 (19.9) |  |  |
| Rent-from local authority, local council, housing association | 2385 (10.4) | 947 (9.6) |  |  |
| **Number of vehicles in household, n(%)** | | | 0.909 | 0.009 |
| One | 11601 (50.4) | 5124 (51.9) |  |  |
| Two | 7600 (33.0) | 3219 (32.6) |  |  |
| None | 2534 (11.0) | 981 (9.9) |  |  |
| Three | 1296 (5.6) | 546 (5.5) |  |  |
| **Average total household income before tax, n(%)** | | | 0.329 | 0.029 |
| 18,000 to 30,999 | 6183 (26.8) | 2658 (26.9) |  |  |
| 31,000 to 51,999 | 7874 (34.2) | 3343 (33.9) |  |  |
| 52,000 to 100,000 | 2201 (9.6) | 965 (9.8) |  |  |
| Prefer not to answer | 1921 (8.3) | 797 (8.1) |  |  |
| Less than 18,000 | 3872 (16.8) | 1683 (17.1) |  |  |
| Greater than 100,000 | 980 (4.3) | 424 (4.3) |  |  |
| **Frequency of friend/family visits, n(%)** | | | 0.317 | 0.029 |
| 1 | 3382 (14.7) | 1410 (14.3) |  |  |
| 2 | 7748 (33.6) | 3398 (34.4) |  |  |
| 3 | 1469 (6.4) | 620 (6.3) |  |  |
| 4 | 7696 (33.4) | 3323 (33.7) |  |  |
| 5 | 2736 (11.9) | 1119 (11.3) |  |  |
| **Able to confide Social support, n(%)** | | | 0.811 | 0.021 |
| Do not know | 726 (3.2) | 304 (3.1) |  |  |
| Once every few months | 1243 (5.4) | 566 (5.7) |  |  |
| About once a week | 2122 (9.2) | 889 (9.0) |  |  |
| Never or almost never | 4311 (18.7) | 1845 (18.7) |  |  |
| Almost daily | 11883 (51.6) | 5128 (52.0) |  |  |
| 2-4 times a week | 1622 (7.0) | 706 (7.2) |  |  |
| About once a month | 1124 (4.9) | 432 (4.4) |  |  |
| **Breastfed as a baby, n(%)** |  |  | 0.491 | 0.014 |
| Yes | 12113 (52.6) | 5180 (52.5) |  |  |
| Do not know | 7692 (33.4) | 3261 (33.0) |  |  |
| No | 3226 (14.0) | 1429 (14.5) |  |  |
| **Irritability, n(%)** |  |  | 0.047 | 0.024 |
| No | 15779 (68.5) | 6701 (67.9) |  |  |
| Do not know | 1026 (4.5) | 406 (4.1) |  |  |
| Yes | 6226 (27.0) | 2763 (28.0) |  |  |
| **Weight change, n(%)** |  |  | 0.966 | 0.003 |
| Yes - lost weight | 3688 (16.0) | 1611 (16.3) |  |  |
| Yes - gained weight | 6368 (27.6) | 2733 (27.7) |  |  |
| No - weigh about the same | 12975 (56.3) | 5526 (56.0) |  |  |
| **TyG, mean (SD)** | 8.97 (0.59) | 8.96 (0.60) | 0.928 | 0.001 |
| **TyG-WHtR, mean (SD)** | 5.17 (0.81) | 5.16 (0.82) | 0.926 | 0.001 |
| **TyG-WC, mean (SD)** | 883.71 (142.54) | 882.57 (144.72) | 0.907 | 0.001 |
| **SIRI, mean (SD)** | 1.42 (1.03) | 1.48 (3.51) | 0.128 | 0.015 |
| **NLR, mean (SD)** | 2.62 (1.50) | 2.67 (2.33) | 0.126 | 0.017 |
| **dNLR, mean (SD)** | 0.85 (0.06) | 0.85 (0.06) | 0.459 | 0.009 |
| **PLR, mean (SD)** | 133.34 (58.67) | 135.05 (108.78) | 0.534 | 0.007 |
| **MLR, mean (SD)** | 0.30 (0.19) | 0.31 (0.73) | 0.225 | 0.012 |
| **NPR, mean (SD)** | 0.02 (0.01) | 0.02 (0.01) | 0.089 | 0.02 |
| **CLR, mean (SD)** | 1.92 (4.21) | 1.93 (4.10) | 0.959 | 0.001 |
| **PIV, mean (SD)** | 344.78 (279.92) | 359.32 (876.62) | 0.188 | 0.013 |
| **IBI, mean (SD)** | 9.91 (25.52) | 10.16 (26.38) | 0.91 | 0.001 |
| **LAP, mean (SD)** | 77.06 (58.12) | 76.59 (58.22) | 0.776 | 0.003 |
| **VAI, mean (SD)** | 2.66 (2.01) | 2.64 (1.96) | 0.841 | 0.002 |
| **CVAI, mean (SD)** | 689.74 (53.95) | 689.30 (54.56) | 0.823 | 0.003 |
| **CTI, mean (SD)** | 9.22 (0.79) | 9.21 (0.79) | 0.224 | 0.014 |
| **eGDR, mean (SD)** | 10.82 (2.11) | 10.88 (2.09) | 0.56 | 0.007 |
| **NON-HDL-C-HDL-C-ratio, mean (SD)** | 3.36 (1.25) | 3.36 (1.25) | 1 | <0.001 |
| **TyG-BMI, mean (SD)** | 260.26 (50.09) | 259.81 (50.96) | 0.851 | 0.002 |
| **Townsend deprivation index, mean (SD)** | -1.06 (3.19) | -1.00 (3.20) | 0.976 | <0.001 |
| **Physical activity, min/week, mean (SD)** | 2708.76 (2763.74) | 2601.32 (2684.00) | 0.766 | 0.004 |
| **BG, mmol/L , mean (SD)** | 5.53 (1.85) | 5.54 (1.90) | 0.999 | <0.001 |
| **HbA1c, mmol/mol, mean (SD)** | 39.52 (9.86) | 39.58 (9.46) | 0.371 | 0.011 |
| **DBP, mmHg, mean (SD)** | 83.80 (11.58) | 83.63 (11.73) | 0.323 | 0.012 |
| **SBP, mmHg, mean (SD)** | 149.04 (22.61) | 148.95 (22.76) | 0.991 | <0.001 |
| **HDL, mmol/L, mean (SD)** | 1.24 (0.32) | 1.24 (0.32) | 0.471 | 0.009 |
| **LDL, mmol/L, mean (SD)** | 2.26 (0.80) | 2.28 (0.82) | 0.164 | 0.017 |
| **Hb, g/L, mean (SD)** | 14.57 (1.29) | 14.57 (1.29) | 0.972 | <0.001 |
| **RDW, %, mean (SD)** | 91.77 (4.78) | 91.75 (4.79) | 0.647 | 0.005 |
| **LYM, *10^9^/L, mean (SD)** | 2.03 (1.34) | 2.03 (1.68) | 0.41 | 0.01 |
| **NE, *10^9^/L, mean (SD)** | 4.69 (1.54) | 4.70 (1.56) | 0.169 | 0.016 |
| **Ala, mmol/L, mean (SD)** | 25.66 (14.33) | 25.92 (15.30) | 0.68 | 0.005 |
| **AST, U/L, mean (SD)** | 27.82 (11.11) | 28.04 (12.57) | 0.869 | 0.002 |
| **DBIL, umol/L, mean (SD)** | 1.95 (0.95) | 1.96 (0.88) | 0.77 | 0.004 |
| **TBIL, umol/L, mean (SD)** | 9.46 (4.50) | 9.58 (4.61) | 0.979 | <0.001 |
| **ALP, U/L, mean (SD)** | 88.46 (28.49) | 87.96 (29.69) | 0.762 | 0.004 |
| **TP, g/L, mean (SD)** | 1.14 (0.17) | 1.14 (0.17) | 0.964 | 0.001 |
| **ALB, g/L, mean (SD)** | 44.89 (2.75) | 44.89 (2.69) | 0.873 | 0.002 |
| **UA, umol/L, mean (SD)** | 352.41 (83.84) | 353.31 (84.02) | 0.876 | 0.002 |
| **eGFR, ml/min/1.73m^2^ , mean (SD)** | 84.54 (17.53) | 84.40 (17.36) | 0.107 | 0.019 |
| **Potassium intake, mg/24h, mean (SD)** | 64.38 (32.53) | 65.01 (33.00) | 0.668 | 0.005 |

Note: *P*: values were calculated using Pearson’ s chi-square test or Fisher’ s exact test for categorical variables. A two-sided P < 0.05 was considered statistically significant. SMD, standardized mean difference. Given the exceptionally large sample size, SMD was utilized to evaluate cohort equilibrium, where an SMD < 0.10 indicates a negligible difference between the training and testing datasets.

**Table S9. Identification of Independent Predictors for RBD Based on Multivariate Logistic Regression.**

| **name** |  | **No (N=9550)** | **Yes (N=320)** | **OR (univariable)** | **OR (multivariable)** |
| --- | --- | --- | --- | --- | --- |
| **Sex** | Male | 7437 (77.9%) | 241 (75.3%) |  |  |
|  | Female | 2113 (22.1%) | 79 (24.7%) | 1.15 (0.89-1.49, *p*=.279) |  |
| **Income** | Income before tax ≤52,000 EUR | 8486 (88.9%) | 302 (94.4%) |  |  |
|  | Income before tax >52,000 EUR | 1064 (11.1%) | 18 (5.6%) | 0.48 (0.29-0.77, *p*=.002) | 0.62 (0.26-1.49, *p*=.284) |
| **Qualification** | Not College or university degree | 7483 (78.4%) | 291 (90.9%) |  |  |
|  | College or university degree | 2067 (21.6%) | 29 (9.1%) | 0.36 (0.25-0.53, *p*<.001) | 0.49 (0.33-0.74, *p*<.001) |
| **Ethnic** | white | 760 (8%) | 21 (6.6%) |  |  |
|  | Other | 8790 (92%) | 299 (93.4%) | 1.23 (0.79-1.93, *p*=.364) |  |
| **Drinking** | No | 917 (9.6%) | 34 (10.6%) |  |  |
|  | Yes | 8633 (90.4%) | 286 (89.4%) | 0.89 (0.62-1.28, *p*=.542) |  |
| **Cancer of family history** | Yes | 5018 (52.5%) | 141 (44.1%) |  |  |
|  | Yes | 4532 (47.5%) | 179 (55.9%) | 1.41 (1.12-1.76, *p*=.003) | 1.31 (1.04-1.65, *p*=.020) |
| **Skin colour** | Fair | 7005 (73.4%) | 229 (71.6%) |  |  |
|  | Light olive | 1558 (16.3%) | 53 (16.6%) | 1.04 (0.77-1.41, *p*=.797) |  |
|  | Very fair | 718 (7.5%) | 29 (9.1%) | 1.24 (0.83-1.83, *p*=.293) |  |
|  | Brown | 269 (2.8%) | 9 (2.8%) | 1.02 (0.52-2.01, *p*=.947) |  |
| **Milk type used** | Semi-skimmed | 6508 (68.1%) | 220 (68.8%) |  |  |
|  | Skimmed | 1983 (20.8%) | 53 (16.6%) | 0.79 (0.58-1.07, *p*=.130) | 0.86 (0.63-1.17, *p*=.339) |
|  | Soya | 295 (3.1%) | 9 (2.8%) | 0.90 (0.46-1.78, *p*=.766) | 0.86 (0.43-1.71, *p*=.671) |
|  | Full cream | 764 (8%) | 38 (11.9%) | 1.47 (1.03-2.09, *p*=.032) | 1.14 (0.79-1.64, *p*=.490) |
| **Processed meat intake** | 2-4 times a week | 2449 (25.6%) | 65 (20.3%) |  |  |
|  | Once a week | 2915 (30.5%) | 101 (31.6%) | 1.31 (0.95-1.79, *p*=.099) | 1.23 (0.89-1.69, *p*=.214) |
|  | Less than once a week | 3263 (34.2%) | 120 (37.5%) | 1.39 (1.02-1.88, *p*=.037) | 1.32 (0.96-1.80, *p*=.084) |
|  | Never | 527 (5.5%) | 17 (5.3%) | 1.22 (0.71-2.09, *p*=.481) | 1.18 (0.68-2.04, *p*=.558) |
|  | 5-6 times a week | 396 (4.1%) | 17 (5.3%) | 1.62 (0.94-2.79, *p*=.083) | 1.37 (0.78-2.39, *p*=.271) |
| **Insomnia** | Usually | 3044 (31.9%) | 107 (33.4%) |  |  |
|  | Sometimes | 4299 (45%) | 149 (46.6%) | 0.99 (0.77-1.27, *p*=.913) |  |
|  | Never/rarely | 2207 (23.1%) | 64 (20%) | 0.82 (0.60-1.13, *p*=.231) |  |
| **Daytime dozing** | Usually | 6404 (67.1%) | 228 (71.2%) |  |  |
|  | Never/rarely | 459 (4.8%) | 12 (3.8%) | 0.73 (0.41-1.32, *p*=.303) |  |
| **Length of mobile phone used** | Five to eight years | 2572 (26.9%) | 75 (23.4%) |  |  |
|  | Two to four years | 2074 (21.7%) | 85 (26.6%) | 1.41 (1.02-1.93, *p*=.035) | 1.37 (0.98-1.89, *p*=.062) |
|  | More than eight years | 2951 (30.9%) | 81 (25.3%) | 0.94 (0.68-1.29, *p*=.710) | 1.09 (0.79-1.51, *p*=.611) |
|  | Never used mobile phone at least once per week | 1668 (17.5%) | 66 (20.6%) | 1.36 (0.97-1.90, *p*=.075) | 1.21 (0.86-1.71, *p*=.270) |
|  | One year or less | 285 (3%) | 13 (4.1%) | 1.56 (0.86-2.85, *p*=.145) | 1.32 (0.72-2.43, *p*=.372) |
| **Own or rent accommodation lived in Household** | Rent - from private landlord or letting agency | 272 (2.8%) | 15 (4.7%) |  |  |
|  | Own outright (by you or someone in your household) | 6472 (67.8%) | 196 (61.2%) | 0.55 (0.32-0.94, *p*=.029) | 0.59 (0.34-1.05, *p*=.072) |
|  | Own with a mortgage | 1909 (20%) | 59 (18.4%) | 0.56 (0.31-1.00, *p*=.051) | 0.78 (0.43-1.43, *p*=.429) |
|  | Rent - from local authority, local council, housing association | 897 (9.4%) | 50 (15.6%) | 1.01 (0.56-1.83, *p*=.972) | 0.69 (0.37-1.27, *p*=.231) |
| **Number of vehicles in household** | One | 4960 (51.9%) | 164 (51.2%) |  |  |
|  | Two | 3127 (32.7%) | 92 (28.8%) | 0.89 (0.69-1.15, *p*=.377) | 1.24 (0.94-1.65, *p*=.133) |
|  | None | 925 (9.7%) | 56 (17.5%) | 1.83 (1.34-2.50, *p*<.001) | 1.28 (0.91-1.82, *p*=.158) |
|  | Three | 538 (5.6%) | 8 (2.5%) | 0.45 (0.22-0.92, *p*=.028) | 0.68 (0.33-1.42, *p*=.305) |
| **Average total household income before tax** | 18,000 to 30,999 | 2576 (27%) | 82 (25.6%) |  |  |
|  | 31,000 to 51,999 | 3196 (33.5%) | 147 (45.9%) | 1.44 (1.10-1.90, *p*=.009) | 1.10 (0.81-1.48, *p*=.549) |
|  | 52,000 to 100,000 | 936 (9.8%) | 29 (9.1%) | 0.97 (0.63-1.50, *p*=.902) | 0.97 (0.62-1.52, *p*=.878) |
|  | Prefer not to answer | 785 (8.2%) | 12 (3.8%) | 0.48 (0.26-0.88, *p*=.019) | 1.22 (0.42-3.60, *p*=.714) |
|  | Less than 18,000 | 1653 (17.3%) | 30 (9.4%) | 0.57 (0.37-0.87, *p*=.009) | 0.70 (0.45-1.08, *p*=.106) |
|  | Greater than 100,000 | 404 (4.2%) | 20 (6.2%) | 1.56 (0.94-2.56, *p*=.083) | 1.19 (0.70-2.01, *p*=.526) |
| **Frequency of friend/family visits** | 1 | 1356 (14.2%) | 54 (16.9%) |  |  |
|  | 2 | 3296 (34.5%) | 102 (31.9%) | 0.78 (0.56-1.09, *p*=.141) |  |
|  | 3 | 605 (6.3%) | 15 (4.7%) | 0.62 (0.35-1.11, *p*=.109) |  |
|  | 4 | 3205 (33.6%) | 118 (36.9%) | 0.92 (0.67-1.28, *p*=.639) |  |
|  | 5 | 1088 (11.4%) | 31 (9.7%) | 0.72 (0.46-1.12, *p*=.144) |  |
| **Able to confide social support** | Do not know | 297 (3.1%) | 7 (2.2%) |  |  |
|  | Once every few months | 546 (5.7%) | 20 (6.2%) | 1.55 (0.65-3.72, *p*=.322) |  |
|  | About once a week | 856 (9%) | 33 (10.3%) | 1.64 (0.72-3.74, *p*=.243) |  |
|  | Never or almost never | 1774 (18.6%) | 71 (22.2%) | 1.70 (0.77-3.73, *p*=.187) |  |
|  | Almost daily | 4975 (52.1%) | 153 (47.8%) | 1.30 (0.61-2.81, *p*=.496) |  |
|  | 2-4 times a week | 682 (7.1%) | 24 (7.5%) | 1.49 (0.64-3.50, *p*=.357) |  |
|  | About once a month | 420 (4.4%) | 12 (3.8%) | 1.21 (0.47-3.12, *p*=.689) |  |
| **Breastfed as a baby** | Yes | 5012 (52.5%) | 168 (52.5%) |  |  |
|  | Do not know | 3158 (33.1%) | 103 (32.2%) | 0.97 (0.76-1.25, *p*=.830) |  |
|  | No | 1380 (14.5%) | 49 (15.3%) | 1.06 (0.77-1.46, *p*=.727) |  |
| **Irritability** | No | 6495 (68%) | 206 (64.4%) |  |  |
|  | Do not know | 390 (4.1%) | 16 (5%) | 1.29 (0.77-2.17, *p*=.331) |  |
|  | Yes | 2665 (27.9%) | 98 (30.6%) | 1.16 (0.91-1.48, *p*=.236) |  |
| **Weight change** | Yes - lost weight | 1564 (16.4%) | 47 (14.7%) |  |  |
|  | Yes - gained weight | 2632 (27.6%) | 101 (31.6%) | 1.28 (0.90-1.82, *p*=.173) |  |
|  | No - weigh about the same | 5354 (56.1%) | 172 (53.8%) | 1.07 (0.77-1.48, *p*=.690) |  |
| **Age** | Mean ± SD | 63.2 ± 5.6 | 64.2 ± 4.2 | 1.03 (1.01-1.06, *p*=.003) | 1.04 (1.01-1.06, *p*=.005) |
| **TyG** | Mean ± SD | 9.0 ± 0.6 | 8.9 ± 0.6 | 0.93 (0.77-1.13, *p*=.480) |  |
| **TyG-WHtR** | Mean ± SD | 5.2 ± 0.8 | 5.1 ± 0.8 | 0.97 (0.84-1.11, *p*=.616) |  |
| **TyG-WC** | Mean ± SD | 882.4 ± 142.7 | 875.1 ± 141.0 | 1.00 (1.00-1.00, *p*=.370) |  |
| **SIRI** | Mean ± SD | 1.5 ± 3.4 | 1.5 ± 0.9 | 1.00 (0.98-1.03, *p*=.753) |  |
| **NLR** | Mean ± SD | 2.7 ± 2.2 | 2.7 ± 1.2 | 1.00 (0.96-1.05, *p*=.922) |  |
| **dNLR** | Mean ± SD | 0.9 ± 0.1 | 0.9 ± 0.1 | 14.76 (1.50-144.80, *p*=.021) | 1.43 (0.08-24.55, *p*=.804) |
| **PLR** | Mean ± SD | 135.5 ± 105.0 | 130.0 ± 55.4 | 1.00 (1.00-1.00, *p*=.188) |  |
| **MLR** | Mean ± SD | 0.3 ± 0.7 | 0.3 ± 0.2 | 0.75 (0.34-1.65, *p*=.471) |  |
| **NPR** | Mean ± SD | 0.0 ± 0.0 | 0.0 ± 0.0 | 38.14 (0.36-4009.69, *p*=.125) |  |
| **CLR** | Mean ± SD | 1.9 ± 4.6 | 2.1 ± 2.6 | 1.00 (0.98-1.02, *p*=.677) |  |
| **PIV** | Mean ± SD | 359.6 ± 855.0 | 395.1 ± 275.8 | 1.00 (1.00-1.00, *p*=.502) |  |
| **IBI** | Mean ± SD | 10.3 ± 28.6 | 11.6 ± 16.5 | 1.00 (1.00-1.00, *p*=.444) |  |
| **LAP** | Mean ± SD | 76.9 ± 58.3 | 74.0 ± 50.6 | 1.00 (1.00-1.00, *p*=.373) |  |
| **VAI** | Mean ± SD | 2.7 ± 2.0 | 2.6 ± 1.7 | 0.99 (0.93-1.05, *p*=.699) |  |
| **CVAI** | Mean ± SD | 689.0 ± 53.7 | 687.5 ± 53.0 | 1.00 (1.00-1.00, *p*=.620) |  |
| **CTI** | Mean ± SD | 9.2 ± 0.8 | 9.3 ± 0.8 | 1.20 (1.05-1.38, *p*=.010) | 1.33 (1.04-1.71, *p*=.021) |
| **eGDR** | Mean ± SD | 10.8 ± 2.1 | 11.0 ± 2.0 | 1.03 (0.97-1.08, *p*=.320) |  |
| **NON-HDL-C-HDL-C-ratio** | Mean ± SD | 3.4 ± 1.3 | 3.3 ± 1.2 | 0.97 (0.88-1.06, *p*=.480) |  |
| **TyG-BMI** | Mean ± SD | 259.8 ± 50.3 | 254.0 ± 49.0 | 1.00 (1.00-1.00, *p*=.042) | 0.99 (0.98-1.00, *p*=.025) |
| **Townsend** | Mean ± SD | -1.1 ± 3.1 | -0.3 ± 3.4 | 1.08 (1.05-1.12, *p*<.001) | 1.04 (0.99-1.08, *p*=.084) |
| **Physical activity** | Mean ± SD | 2657.8 ± 2703.3 | 2767.9 ± 3150.6 | 1.00 (1.00-1.00, *p*=.476) |  |
| **Blood Glucose** | Mean ± SD | 5.5 ± 1.9 | 5.5 ± 1.7 | 0.99 (0.93-1.05, *p*=.641) |  |
| **HbA1c** | Mean ± SD | 39.6 ± 9.6 | 40.3 ± 8.6 | 1.01 (1.00-1.02, *p*=.200) | 1.05(1.03-1.07, *p*=.239) |
| **DBP** | Mean ± SD | 83.7 ± 11.7 | 82.3 ± 10.9 | 0.99 (0.98-1.00, *p*=.029) | 0.99 (0.98-1.00, *p*=.181) |
| **SBP** | Mean ± SD | 149.0 ± 22.9 | 146.7 ± 21.4 | 1.00 (0.99-1.00, *p*=.074) |  |
| **HDL-C** | Mean ± SD | 1.2 ± 0.3 | 1.2 ± 0.3 | 0.90 (0.63-1.28, *p*=.541) |  |
| **LDL-C** | Mean ± SD | 2.3 ± 0.8 | 2.2 ± 0.8 | 0.90 (0.78-1.03, *p*=.133) |  |
| **Hb** | Mean ± SD | 14.6 ± 1.3 | 14.7 ± 1.4 | 1.06 (0.97-1.16, *p*=.186) |  |
| **RDW** | Mean ± SD | 91.7 ± 4.8 | 92.1 ± 5.1 | 1.02 (0.99-1.04, *p*=.188) |  |
| **LY** | Mean ± SD | 2.0 ± 1.5 | 2.1 ± 0.7 | 1.03 (0.99-1.07, *p*=.160) |  |
| **NE** | Mean ± SD | 4.7 ± 1.6 | 5.3 ± 1.7 | 1.20 (1.14-1.27, *p*<.001) | 1.13 (1.04-1.21, *p*=.002) |
| **Ala** | Mean ± SD | 26.0 ± 14.9 | 23.1 ± 13.4 | 0.98 (0.97-0.99, *p*<.001) | 0.99 (0.98-1.01, *p*=.226) |
| **AST** | Mean ± SD | 28.0 ± 13.2 | 26.2 ± 8.5 | 0.98 (0.96-0.99, *p*=.003) |  |
| **DBIL** | Mean ± SD | 2.0 ± 0.9 | 1.8 ± 0.7 | 0.80 (0.68-0.93, *p*=.004) | 0.97 (0.77-1.21, *p*=.782) |
| **TBIL** | Mean ± SD | 9.6 ± 4.7 | 8.6 ± 3.4 | 0.94 (0.91-0.97, *p*<.001) | 0.96 (0.92-1.01, *p*=.106) |
| **ALP** | Mean ± SD | 88.1 ± 27.9 | 92.9 ± 24.0 | 1.00 (1.00-1.01, *p*=.003) | 1.00 (1.00-1.01, *p*=.291) |
| **TP** | Mean ± SD | 1.1 ± 0.2 | 1.1 ± 0.2 | 1.49 (0.76-2.90, *p*=.244) |  |
| **ALB** | Mean ± SD | 44.9 ± 2.7 | 44.7 ± 2.9 | 0.98 (0.94-1.02, *p*=.239) |  |
| **UA** | Mean ± SD | 353.7 ± 84.6 | 352.2 ± 86.5 | 1.00 (1.00-1.00, *p*=.753) |  |
| **eGFR** | Mean ± SD | 84.38 (17.19) | 84.49 (16.96) | 1.21 (0.99-1.41, *p*=.787) |  |
| **Potassium intake** | Mean ± SD | 64.9 ± 32.4 | 60.2 ± 33.1 | 1.00 (0.99-1.00, *p*=.010) | 1.00 (0.99-1.00, *p*=.128) |

Note: *P* values were calculated using Pearson’ s chi-square test or Fisher’ s exact test for categorical variables. A two-sided *P* < 0.05 was considered statistically significant.

**Table S10. Collinearity Analysis of Selected Variables.**

| **Variable** | **VIF** (All candidate variables) |
| --- | --- |
| TyG | 9.181226697 |
| PLR | 8.38963722 |
| CLR | 8.387540434 |
| NLR | 8.271828757 |
| IBI | 8.09161441 |
| SIRI | 8.047381296 |
| PIV | 7.773632241 |
| LAP | 6.241160005 |
| MLR | 6.240019141 |
| TyG_WC | 6.198694357 |
| NON_HDL_C_HDL_C_ratio | 5.920772792 |
| VAI | 5.52652659 |
| TyG_WHtR | 5.470916889 |
| TyG_BMI | 3.843125061 |
| NE | 3.78576221 |
| LDL | 3.601756351 |
| CTI | 3.600062 |
| DBIL | 3.117795464 |
| TBIL | 3.106905147 |
| BG | 2.720205442 |
| HDL | 2.548461977 |
| dNLR | 2.429537966 |
| Monocyte count | 2.312273123 |
| ALa | 2.306051982 |
| DBP | 2.206768664 |
| AST | 2.187859267 |
| HbA1c | 2.085068514 |
| SBP | 2.070075566 |
| eGDR | 1.981520929 |
| NPR | 1.914214947 |
| Age | 1.806185 |
| Hb | 1.528892474 |
| LYM | 1.209392958 |
| UA | 1.186908384 |
| ALB | 1.150527847 |
| ALP | 1.14455835 |
| TP | 1.121975369 |
| RDW | 1.101656482 |
| Townsend | 1.061282965 |
| Potassium intake | 1.042562779 |
| Physical activity | 1.017599916 |
| eGFR | 1.001082548 |

**Table S11. Performance of eight machine learning models in the training and internal test sets.**

| **Data set** | **Model** | **Threshold** | **Accuracy** | **Sensitivity** | **Specificity** | **Precision** | **F1** |
| --- | --- | --- | --- | --- | --- | --- | --- |
| **Train** | Logistic | 0.509 | 0.650 | 0.649 | 0.652 | 0.651 | 0.650 |
|  | SVM | 0.558 | 0.653 | 0.639 | 0.666 | 0.657 | 0.648 |
|  | GBM | 0.510 | 0.784 | 0.736 | 0.831 | 0.814 | 0.773 |
|  | NeuralNetwork | 0.494 | 0.677 | 0.726 | 0.629 | 0.662 | 0.692 |
|  | Xgboost | 0.500 | 0.685 | 0.728 | 0.643 | 0.671 | 0.698 |
|  | Adaboost | 0.759 | 0.645 | 0.521 | 0.768 | 0.692 | 0.594 |
|  | LightGBM | 0.513 | 0.791 | 0.725 | 0.857 | 0.836 | 0.776 |
|  | CatBoost | 0.627 | 0.746 | 0.695 | 0.796 | 0.773 | 0.732 |
| **Test** | Logistic | 0.422 | 0.649 | 0.819 | 0.480 | 0.611 | 0.700 |
|  | SVM | 0.572 | 0.641 | 0.608 | 0.675 | 0.652 | 0.629 |
|  | GBM | 0.505 | 0.727 | 0.695 | 0.758 | 0.742 | 0.718 |
|  | NeuralNetwork | 0.466 | 0.657 | 0.747 | 0.567 | 0.633 | 0.685 |
|  | Xgboost | 0.500 | 0.645 | 0.727 | 0.564 | 0.625 | 0.672 |
|  | Adaboost | 0.241 | 0.580 | 0.906 | 0.253 | 0.548 | 0.683 |
|  | LightGBM | 0.603 | 0.697 | 0.547 | 0.847 | 0.781 | 0.643 |
|  | CatBoost | 0.635 | 0.699 | 0.536 | 0.863 | 0.796 | 0.641 |

**Table S12. Pairwise DeLong Test Z-statistics and P-values for AUC Comparisons Among the Eight Machine Learning Models.**

| **Data Set** | **Model 1** | **Model 2** | **AUC 1** | **AUC 2** | **AUC Difference** | **Z-Statistic** | **P-Value** |
| --- | --- | --- | --- | --- | --- | --- | --- |
| **Train** | Logistic | GBM | 0.6984 | 0.8589 | -0.1604 | -22.1961 | 0 |
|  | Logistic | NeuralNetwork | 0.6984 | 0.7333 | -0.0349 | -5.173 | 0 |
|  | Logistic | Xgboost | 0.6984 | 0.7456 | -0.0471 | -6.3493 | 0 |
|  | Logistic | LightGBM | 0.6984 | 0.8663 | -0.1679 | -18.1779 | 0 |
|  | Logistic | CatBoost | 0.6984 | 0.8285 | -0.1301 | -16.9449 | 0 |
|  | SVM | GBM | 0.6955 | 0.8589 | -0.1634 | -22.4301 | 0 |
|  | SVM | NeuralNetwork | 0.6955 | 0.7333 | -0.0379 | -5.4999 | 0 |
|  | SVM | Xgboost | 0.6955 | 0.7456 | -0.0501 | -7.6663 | 0 |
|  | SVM | LightGBM | 0.6955 | 0.8663 | -0.1708 | -18.404 | 0 |
|  | SVM | CatBoost | 0.6955 | 0.8285 | -0.133 | -18.2529 | 0 |
|  | GBM | NeuralNetwork | 0.8589 | 0.7333 | 0.1255 | 17.996 | 0 |
|  | GBM | Xgboost | 0.8589 | 0.7456 | 0.1133 | 17.5103 | 0 |
|  | GBM | Adaboost | 0.8589 | 0.6744 | 0.1845 | 22.8607 | 0 |
|  | GBM | CatBoost | 0.8589 | 0.8285 | 0.0304 | 7.3389 | 0 |
|  | NeuralNetwork | Adaboost | 0.7333 | 0.6744 | 0.0589 | 6.6911 | 0 |
|  | NeuralNetwork | LightGBM | 0.7333 | 0.8663 | -0.133 | -15.0133 | 0 |
|  | NeuralNetwork | CatBoost | 0.7333 | 0.8285 | -0.0952 | -12.6124 | 0 |
|  | Xgboost | Adaboost | 0.7456 | 0.6744 | 0.0712 | 9.719 | 0 |
|  | Xgboost | LightGBM | 0.7456 | 0.8663 | -0.1208 | -14.3486 | 0 |
|  | Xgboost | CatBoost | 0.7456 | 0.8285 | -0.083 | -14.513 | 0 |
|  | Adaboost | LightGBM | 0.6744 | 0.8663 | -0.1919 | -20.8791 | 0 |
|  | Adaboost | CatBoost | 0.6744 | 0.8285 | -0.1541 | -18.7716 | 0 |
|  | LightGBM | CatBoost | 0.8663 | 0.8285 | 0.0378 | 5.746 | 0 |
|  | GBM | LightGBM | 0.8589 | 0.8663 | -0.0074 | -1.2949 | 0 |
|  | Logistic | Adaboost | 0.6984 | 0.6744 | 0.024 | 2.71 | 0.0067 |
|  | SVM | Adaboost | 0.6955 | 0.6744 | 0.0211 | 2.4886 | 0.0128 |
|  | NeuralNetwork | Xgboost | 0.7333 | 0.7456 | -0.0122 | -1.565 | 0.1176 |
|  | Logistic | SVM | 0.6984 | 0.6955 | 0.0029 | 1.0527 | 0.2925 |
| **Test** | Logistic | GBM | 0.6904 | 0.8015 | -0.1111 | -9.8082 | 0 |
|  | Logistic | Adaboost | 0.6904 | 0.615 | 0.0754 | 5.342 | 0 |
|  | Logistic | CatBoost | 0.6904 | 0.7733 | -0.0829 | -6.5414 | 0 |
|  | SVM | GBM | 0.6779 | 0.8015 | -0.1236 | -10.9216 | 0 |
|  | SVM | Adaboost | 0.6779 | 0.615 | 0.0629 | 4.6896 | 0 |
|  | SVM | LightGBM | 0.6779 | 0.7449 | -0.067 | -4.2701 | 0 |
|  | SVM | CatBoost | 0.6779 | 0.7733 | -0.0954 | -8.0546 | 0 |
|  | GBM | NeuralNetwork | 0.8015 | 0.6889 | 0.1126 | 9.6785 | 0 |
|  | GBM | Xgboost | 0.8015 | 0.6933 | 0.1082 | 9.9771 | 0 |
|  | GBM | Adaboost | 0.8015 | 0.615 | 0.1865 | 14.0921 | 0 |
|  | GBM | LightGBM | 0.8015 | 0.7449 | 0.0566 | 5.15 | 0 |
|  | NeuralNetwork | Adaboost | 0.6889 | 0.615 | 0.0739 | 5.2248 | 0 |
|  | NeuralNetwork | CatBoost | 0.6889 | 0.7733 | -0.0844 | -6.6147 | 0 |
|  | Xgboost | Adaboost | 0.6933 | 0.615 | 0.0783 | 6.4651 | 0 |
|  | Xgboost | CatBoost | 0.6933 | 0.7733 | -0.08 | -8.4476 | 0 |
|  | Adaboost | LightGBM | 0.615 | 0.7449 | -0.1298 | -7.8733 | 0 |
|  | Adaboost | CatBoost | 0.615 | 0.7733 | -0.1583 | -11.7836 | 0 |
|  | GBM | CatBoost | 0.8015 | 0.7733 | 0.0282 | 3.848 | 0.0001 |
|  | NeuralNetwork | LightGBM | 0.6889 | 0.7449 | -0.056 | -3.5852 | 0.0003 |
|  | Logistic | LightGBM | 0.6904 | 0.7449 | -0.0544 | -3.4982 | 0.0005 |
|  | Xgboost | LightGBM | 0.6933 | 0.7449 | -0.0516 | -3.3649 | 0.0008 |
|  | Logistic | SVM | 0.6904 | 0.6779 | 0.0125 | 2.79 | 0.0053 |
|  | LightGBM | CatBoost | 0.7449 | 0.7733 | -0.0284 | -2.2466 | 0.0247 |
|  | SVM | Xgboost | 0.6779 | 0.6933 | -0.0154 | -1.4818 | 0.1384 |
|  | SVM | NeuralNetwork | 0.6779 | 0.6889 | -0.011 | -0.9682 | 0.333 |
|  | NeuralNetwork | Xgboost | 0.6889 | 0.6933 | -0.0044 | -0.3343 | 0.7382 |
|  | Logistic | Xgboost | 0.6904 | 0.6933 | -0.0029 | -0.2361 | 0.8133 |
|  | Logistic | NeuralNetwork | 0.6904 | 0.6889 | 0.0016 | 0.1355 | 0.8922 |

**Figure S1. Flowchart for the selection of the study population from the UK Biobank study.**

**Figure S2. Comulative risk of cancer.**

**References**

[1] Inker LA, Eneanya ND, Coresh J, et al. New Creatinine- and Cystatin C-Based Equations

to Estimate GFR without Race. N Engl J Med. 2021;385:1737-1749.

[2] SCORE2 working group and ESC Cardiovascular risk collaboration. “SCORE2 risk prediction algorithms: new models to estimate 10-year risk of cardiovascular disease in Europe.” European heart journal vol. 42,25 (2021): 2439-2454. doi:10.1093/eurheartj/ehab309
